# Male-Biased Aganglionic Megacolon in the TashT Mouse Line Due to Perturbation of Silencer Elements in a Large Gene Desert of Chromosome 10

**DOI:** 10.1371/journal.pgen.1005093

**Published:** 2015-03-18

**Authors:** Karl-F. Bergeron, Tatiana Cardinal, Aboubacrine M. Touré, Mélanie Béland, Diana L. Raiwet, David W. Silversides, Nicolas Pilon

**Affiliations:** 1 Molecular Genetics of Development Laboratory, Department of Biological Sciences and BioMed Research Center, University of Quebec at Montreal (UQAM), Quebec, Canada; 2 Veterinary Genetics Laboratory, Faculty of Veterinary Medicine, University of Montreal, Quebec, Canada; National Institute for Medical Research, United Kingdom

## Abstract

Neural crest cells (NCC) are a transient migratory cell population that generates diverse cell types such as neurons and glia of the enteric nervous system (ENS). Via an insertional mutation screen for loci affecting NCC development in mice, we identified one line—named TashT—that displays a partially penetrant aganglionic megacolon phenotype in a strong male-biased manner. Interestingly, this phenotype is highly reminiscent of human Hirschsprung’s disease, a neurocristopathy with a still unexplained male sex bias. In contrast to the megacolon phenotype, colonic aganglionosis is almost fully penetrant in homozygous TashT animals. The sex bias in megacolon expressivity can be explained by the fact that the male ENS ends, on average, around a “tipping point” of minimal colonic ganglionosis while the female ENS ends, on average, just beyond it. Detailed analysis of embryonic intestines revealed that aganglionosis in homozygous TashT animals is due to slower migration of enteric NCC. The TashT insertional mutation is localized in a gene desert containing multiple highly conserved elements that exhibit repressive activity in reporter assays. RNAseq analyses and 3C assays revealed that the TashT insertion results, at least in part, in NCC-specific relief of repression of the uncharacterized gene Fam162b; an outcome independently confirmed via transient transgenesis. The transcriptional signature of enteric NCC from homozygous TashT embryos is also characterized by the deregulation of genes encoding members of the most important signaling pathways for ENS formation—Gdnf/Ret and Edn3/Ednrb—and, intriguingly, the downregulation of specific subsets of X-linked genes. In conclusion, this study not only allowed the identification of *Fam162b* coding and regulatory sequences as novel candidate loci for Hirschsprung’s disease but also provides important new insights into its male sex bias.

## Author Summary

Hirschsprung’s disease (also known as aganglionic megacolon) is a severe congenital defect of the enteric nervous system (ENS) resulting in complete failure to pass stools. It is characterized by the absence of neural ganglia (aganglionosis) in the distal gut due to incomplete colonization of the embryonic intestines by neural crest cells (NCC), the ENS precursors. Hirschsprung’s disease has an incidence of 1 in 5000 newborns and a 4:1 male sex bias. Although many genes have been associated with this complex genetic disease, most of its heritability as well as its male sex bias remain unexplained. Here, we describe an insertional mutant mouse line (“TashT”) in which virtually all homozygotes display colonic aganglionosis due to defective migration of enteric NCC, but in which only a subset of homozygotes develops megacolon. Surprisingly, this group is almost exclusively male. The TashT ENS defect stems, at least in part, from the disruption of long-range interactions between evolutionarily conserved elements with silencer activity and *Fam162b*, resulting in NCC-specific upregulation of this uncharacterized protein coding gene. Global analysis of gene expression further revealed that several hundreds of genes are significantly deregulated in TashT enteric NCC. Interestingly, this dataset includes multiple X-linked candidate genes potentially underlying the male sex bias. Taken together, our data pave the way for a clearer understanding of the intriguing male sex bias of Hirschsprung’s disease.

## Introduction

The enteric nervous system (ENS) is the intrinsic neural network of the gastrointestinal tract. One of its essential roles is to regulate intestinal motility. The ENS is made up of interconnected neural ganglia, themselves composed of neurons and supporting glial cells, forming two main parallel networks: the submucosal plexus and the myenteric plexus. The muscles of the bowel wall that ensure peristaltic movements are controlled by the myenteric plexus of the ENS.

The ENS is constructed during embryo development by derivatives of migrating neural crest cells (NCC) [1]. These multipotent cells originate from the dorsal part of the neural tube, undergo an epithelial-mesenchymal transition, and migrate extensively to contribute to numerous embryonic structures. Among several different cell types, NCC generate melanocytes as well as all enteric neurons and glia. The developing bowel is mainly colonized by NCC derivatives originating from the vagal region of the neural tube. Such colonization proceeds as a rostro-caudal wave lasting more than 5 days in the mouse (from embryonic day (e) 9.0 to 14.5), with NCC derivatives first entering the foregut, passing through the midgut (prospective small intestine) and finally populating the hindgut (prospective colon) either by migrating through the intestinal mesenchyme [2] or by taking a shortcut via the mesentery [3]. The hindgut is the last part of the intestines to be colonized and, therefore, the most susceptible to enteric NCC (eNCC) developmental defects. Sacral NCC also contribute to the ENS, but this later contribution is minor and cannot compensate for a lack of vagal NCC [4].

Defects in hindgut colonization by eNCC result in a lack of neural ganglia in the colon, leading to intestinal blockage due to absence of peristalsis. This phenotype is generally described as “aganglionic megacolon” because of the subsequent massive accumulation of fecal material and severe distention of the colon. In humans, this condition is called Hirschsprung's disease (HSCR) and, depending on the length of aganglionosis, is clinically subdivided in short-segment (i.e. restricted to the rectosigmoid colon) and long-segment forms. Short-segment HSCR represents the vast majority of cases and is more common in males than females, with an overall ratio of ~4:1 [5]. In patients displaying longer segments of aganglionosis, the sex bias is much less pronounced or absent altogether. Although mutations in at least 15 genes have been implicated in HSCR, heritability is unexplained for the majority of cases [6]. HSCR is thus a classic example of a complex disease involving multiple genes, incomplete penetrance, variable expressivity and an intriguing male bias.

Most known HSCR-associated genes encode players from two signaling pathways: the GDNF ligand/ RET receptor and EDN3 ligand/ EDNRB receptor pathways. In fact, *RET* is the main gene associated with HSCR [5]. For both pathways, the receptor is found at the surface of eNCC while the ligand is dynamically secreted from the surrounding mesenchyme during the colonization phase. The role of GDNF/RET and EDN3/EDNRB signaling in ENS formation has been well conserved evolutionarily and studies in animal models have revealed that both pathways profoundly influence every key aspect of eNCC development such as proliferation, survival, differentiation and, most especially, migration [7]. Mouse models have been particularly informative in this regard and multiple lines bearing mutation—either spontaneous or targeted—of genes encoding members of Gdnf/Ret and Edn3/Ednrb pathways have been studied [8–13]. However, the incomplete penetrance and, above all, the male bias observed in human HSCR have been poorly replicated in current animal models [14].

Here, we report the creation of a new insertional mutant mouse model for HSCR that displays, for the first time, incomplete penetrance of the aganglionic megacolon phenotype with a very strong male bias. Extensive characterization of this mouse line and independent validation via transient transgenesis indicate that this outcome is, at least in part, initiated by the specific upregulation of *Fam162b* in NCC.

## Results

### Phenotypic overview of the TashT mouse line

**Fig 1:**
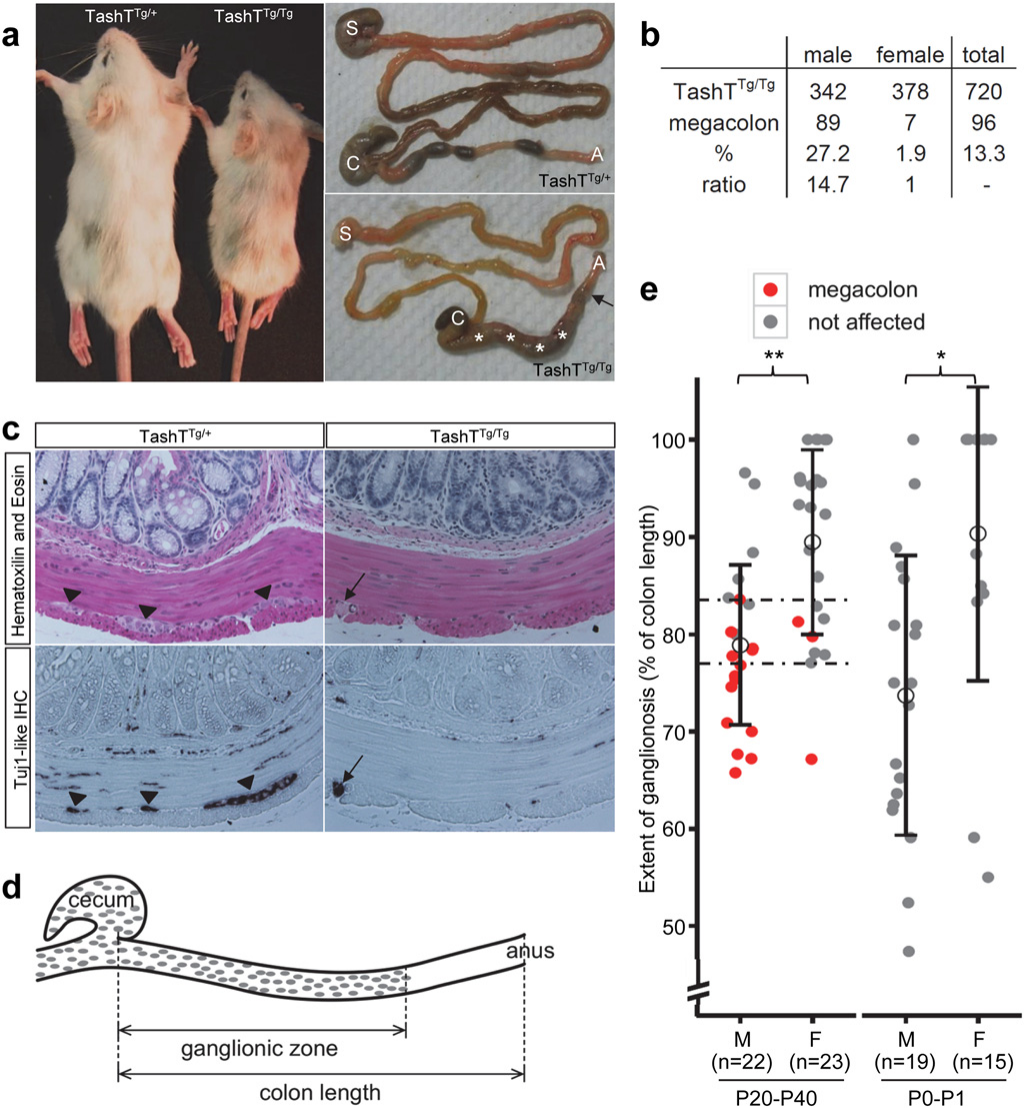
The TashT mouse line is a model for male-biased aganglionic megacolon. (a) Comparison between heterozygous and homozygous TashT animals from a F2 litter at postnatal day (P) 21. Between one and two weeks after birth, a fraction of TashT^Tg/Tg^ mutants exhibit symptoms of aganglionic megacolon such as growth delay (left panel), general weakness and hunched posture. Right panels show that these animals eventually die around weaning age due to complete blockage of the colon (arrow) and accumulation of fecal material (asterisks). S, stomach; C, cecum; A, anus. (b) Table of megacolon incidence in the TashT^Tg/Tg^ colony indicating partial penetrance and strong male bias of the megacolon phenotype. (c) Hematoxylin-Eosin (H&E) staining and IHC of neuron-specific βIII-Tubulin (TuJ1-like) of serial sections of P21 distal colons. The arrowheads in the left panels point to myenteric neural ganglia that can only be detected in TashT^Tg/+^ tissues. The black arrow in the right panels indicates the presence of defasciculated axonal projections within the longitudinal muscle layer of TashT^Tg/Tg^ tissues, another hallmark of aganglionic megacolon. (d) Schematic drawing of the colon of TashT^Tg/Tg^ animals, showing the measurements made for calculating the length of the ganglionic zone of the myenteric plexus as determined by staining of acetylcholinesterase activity. (e) Quantification of the length of the ganglionic zone (in % of total colon length) in the colon of TashT^Tg/Tg^ animals aged between P20 and P40 (left) or between P0 and P1 (right), showing significant differences between males and females according to a t-test (**p = 0.00025; *p = 0.00282). The critical region for developing megacolon (between dashed lines) is delimited by the longest ganglionic zone found in a megacolon case (83.6%) and the shortest ganglionic zone observed in a non-affected individual (77.1%).

The TashT mouse line was obtained from an insertional mutagenesis screen for genes involved in NCC development. This screen was based on the random insertion of a *Tyrosinase* (*Tyr*) minigene in the FVB/n genetic background. Owing to its specific expression in melanocytes, the *Tyr* minigene rescues the albino phenotype of FVB/n mice and thus provides a visible—and generally uniform—pigmentation marker for transgenesis [15]. Since melanocytes are derived from NCC, this genetic tool also proved to be a potent indicator of abnormal NCC development via identification of non-uniform pigmentation patterns. This approach yielded several transgenic mutant lines (to be described elsewhere) among which TashT (*Tachetée*, in French) was identified due to its variegated pigmentation (Fig. 1a).

In addition to the pigmentary anomalies that are similar in both heterozygous and homozygous mutants, a subset of TashT^Tg/Tg^ animals suffer from aganglionic megacolon around weaning age. These animals are smaller than unaffected TashT^Tg/Tg^ siblings (reaching about 74% of littermate weight) and exhibit bowel obstruction concomitant with lack of myenteric ganglia in the distal colon (Fig. 1a,c). The most striking and interesting feature of this lethal phenotype is the fact that the vast majority of affected animals are male (Fig. 1b). In addition, we found via histological analyses that colonic aganglionosis can not only be detected in megacolon-suffering but also in non-affected TashT^Tg/Tg^ animals of both sexes (Fig. 1c). To determine whether megacolon expressivity could be correlated with extent of aganglionosis, we undertook a systematic analysis of the length of the colonic ENS for a random group of TashT^Tg/Tg^ animals of weaning age via staining of acetylcholinesterase activity (Fig. 1d). In accordance with such correlation, quantification results first revealed that—regardless of the sex of the animal—a minimal length of the colon (~ 80%; critical region in Fig. 1e) has to be properly innervated in FVB/n mice in order to avoid blockage. No intermediate phenotype was noted in the course of these analyses as none of the non-affected animals showed signs of abnormal accumulation of feces in the colon. Furthermore, these results confirmed that most TashT^Tg/Tg^ animals exhibit aganglionosis in the distal colon and that males (ganglionated on average over 79% of total colon length) are more affected than females (ganglionated on average over 89% of total colon length) (Fig. 1e). Importantly, a similar statistically significant difference between male and female animals (74% vs 90%) was also observed in neonatal colons, thus confirming the developmental origin of this male-biased defect.

### Detailed analysis of TashT^Tg/Tg^ eNCC reveals a cell migration defect

**Fig 2:**
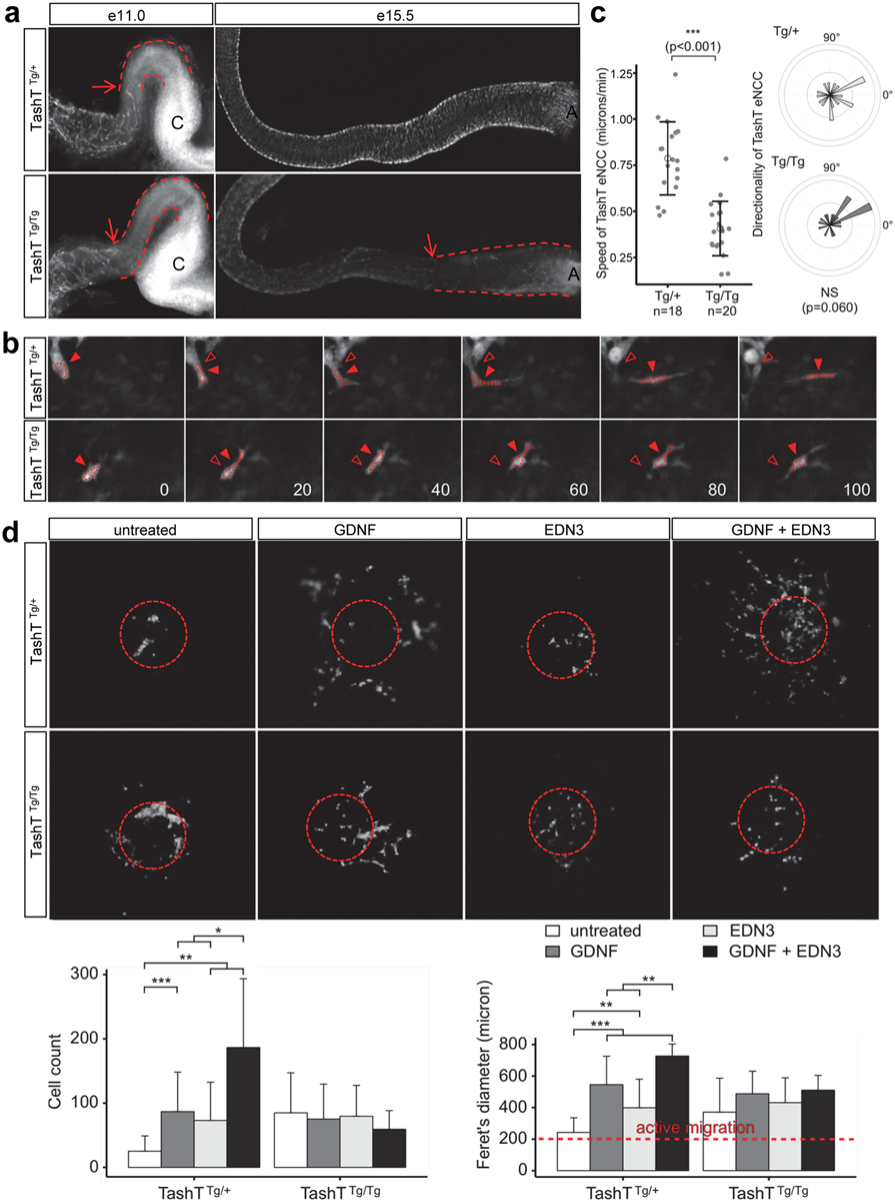
A cell migration defect underlies the defective colonization of TashT^Tg/Tg^ embryonic guts by eNCC. **(a)** Dissected embryonic intestines at e11.0 (left panel) and e15.5 (right panel) show a delay in eNCC colonization in TashT^Tg/Tg^ embryos relative to TashT^Tg/+^. Arrows point to the migration front and dashed lines indicate the distance between eNCC migration front and cecum (C) at e11.0 or anus (A) at e15.5. **(b)** Representative images from time-lapse recordings of eNCC movement at the migration front in e11.0 TashT embryos. Arrowheads point to the centre of an outlined eNCC. Empty arrowheads show the starting position of the cell for easy visualization of the shorter migration distance in TashT^Tg/Tg^ embryos. The time (in minutes) between successive images is shown in the bottom right. **(c)** Quantification of eNCC migration speed shows that eNCC in TashT^Tg/Tg^ embryos are significantly slower than in TashT^Tg/+^ embryos according to a t-test (***: p < 0.001) while directionality is not significantly affected. **(d)** Top panels show representative images of migration assay results obtained with TashT intestinal explants in the presence of GDNF (10 ng/ml), EDN3 (250 ng/ml) and both extracellular ligands. Dotted circles indicate the position of the explant before its removal for imaging. Bottom panels show the quantification of the number of cells that invaded the collagen gel (left) and their spread (Feret's diameter; rigth). A Feret's diameter above 200 microns (the average diameter of intestinal slices used in the assay; red dotted line) indicates active migration away from the explant. For TashT^Tg/+^: untreated, n = 13; GDNF, n = 18; EDN3, n = 15; GDNF + EDN3, n = 6. For TashT^Tg/Tg^: untreated, n = 18; GDNF, n = 8; EDN3, n = 14; GDNF + EDN3, n = 10. Significant differences were found between some treatments according to a t-test (*: *p* < 0.05, **: *p* < 0.01, ***: *p* < 0.001).

To analyze ENS formation in TashT embryos, we took advantage of the fact that this line bears a second co-injected transgene (pSRYp[1.6kb]-YFP) that labels migrating NCC derivatives (including eNCC of vagal and sacral origin) with YFP fluorescence [16] (see also S1 Fig). Whole-mount detection of fluorescence in dissected stage-matched embryonic intestines revealed that, in comparison to TashT^Tg/+^ or G4-GFP control embryos [17] (S2 Fig), a colonization delay by eNCC of vagal origin is clearly observed for TashT^Tg/Tg^ embryos starting around e11.0 (Fig. 2a). Although ectopic fluorescence in the cecum and proximal hindgut regions impeded precise visualization of the migration front between e11.5 and e14.5, we found that this delay persists through the time at which normal colonization of the digestive tract is overtly completed (e15.5). The presence of scattered fluorescent cells beyond the chains of vagal-derived eNCC at this stage also suggests that the contribution of sacral-derived eNCC to the distal hindgut is not abrogated in TashT^Tg/Tg^ embryos.

Closer inspection of the migration front at e11.0 showed that cell protrusions in the form of filopodia were not overtly affected in TashT^Tg/Tg^ eNCC (S3 Fig), suggesting that cells could still investigate, and had the capacity to respond to, their environment. To further characterize the colonization defect, leader cells at the tip of eNCC chains were then visualized during several hours in *ex vivo* cultures of e11.0 intestines from littermate control (TashT^Tg/+)^ and mutant (TashT^Tg/Tg^) embryos (Figs. 2b, S4 and S1–S2 Videos). It is noteworthy that TashT^Tg/Tg^ intestines were selected for these analyses on the basis of the severity of their colonization defect in order to increase the odds of detecting differences between control and mutant eNCC. Using these conditions, we found that the average migration speed, and therefore travel distance, is almost halved in TashT^Tg/Tg^ leader eNCC while directionality of migration is not noticeably affected (Fig. 2c). Given the selection bias towards more affected embryos, it is important to bear in mind that this severe effect is most likely not representative of the true average migration speed of mutant eNCC. It should also be noted that the small difference in eNCC location along the intestines—before cecum (TashT^Tg/Tg^) vs entry of cecum (TashT^Tg/+^)—cannot account for the observed difference in speed since the eNCC migration front normally displays a fairly stable net speed of ~35 micron/hour (~0.58 micron/min) between e10.5 and e12.5 [18].

Migration of eNCC is dependent on multiple signaling pathways among which GDNF/RET and EDN3/EDNRB are recognized as the most critical regulators [19–22]. To evaluate the status of these signaling pathways in TashT embryos, we made use of a recently described quantitative migration assay using e12.5 midgut explants [23]. With control TashT^Tg/+^ or G4-GFP tissues, collagen gels containing either GDNF or EDN3 increased the number of cells coming out of the explants (Fig. 2d). Interestingly, when these ligands were used in combination, a synergistic increase in eNCC numbers invading the collagen was observed. However, little reaction to these extracellular ligands was detected in eNCC derived from TashT^Tg/Tg^ embryos (Fig. 2d). In fact, eNCC from homozygous embryos migrated out of intestinal explants even in the absence of exogenous ligands, suggesting they lost some sensitivity to their endogenous microenvironment and distinguished poorly between intestinal tissue and collagen gel.

Premature differentiation or a scarcity of progenitor cells can disturb eNCC colonization and lead to incomplete ENS formation [19,22,24,25]. To verify whether these processes might contribute to the TashT phenotype, we performed a detailed marker analysis of embryonic intestines in order to quantify neuronal and glial differentiation as well as proliferation and cell death of eNCC. Quantification of proliferation and cell death in e12.5 stage-matched bowel tissues failed to reveal any significant difference between TashT^Tg/Tg^ and control TashT^Tg/+^ embryos (S5 Fig). Assessment of neuronal differentiation at the same stage also failed to reveal any significant difference (S6a-S6b Fig). On the other hand, glial differentiation at e15.5 was found to be less prevalent in TashT^Tg/Tg^ distal bowel tissues, to the benefit of undifferentiated progenitors (S6c-S6d Fig). This, however, is most likely a consequence of the delay in rostro-caudal colonization, and goes contrary to the idea that premature differentiation is the cause of the TashT migration defect. Taken together, these results thus highlight the eNCC migration defect (concomitant with insensitivity towards GDNF and EDN3) as the principal cause of the TashT aganglionosis phenotype. Moreover, since no clear sex bias was observed in these analyses, the relatively modest male bias in phenotype severity is most likely the result of an accumulation of subtle differences during the whole ENS developmental time window.

### The TashT transgenic insertion disrupts evolutionarily conserved regions possessing silencer activity

**Fig 3:**
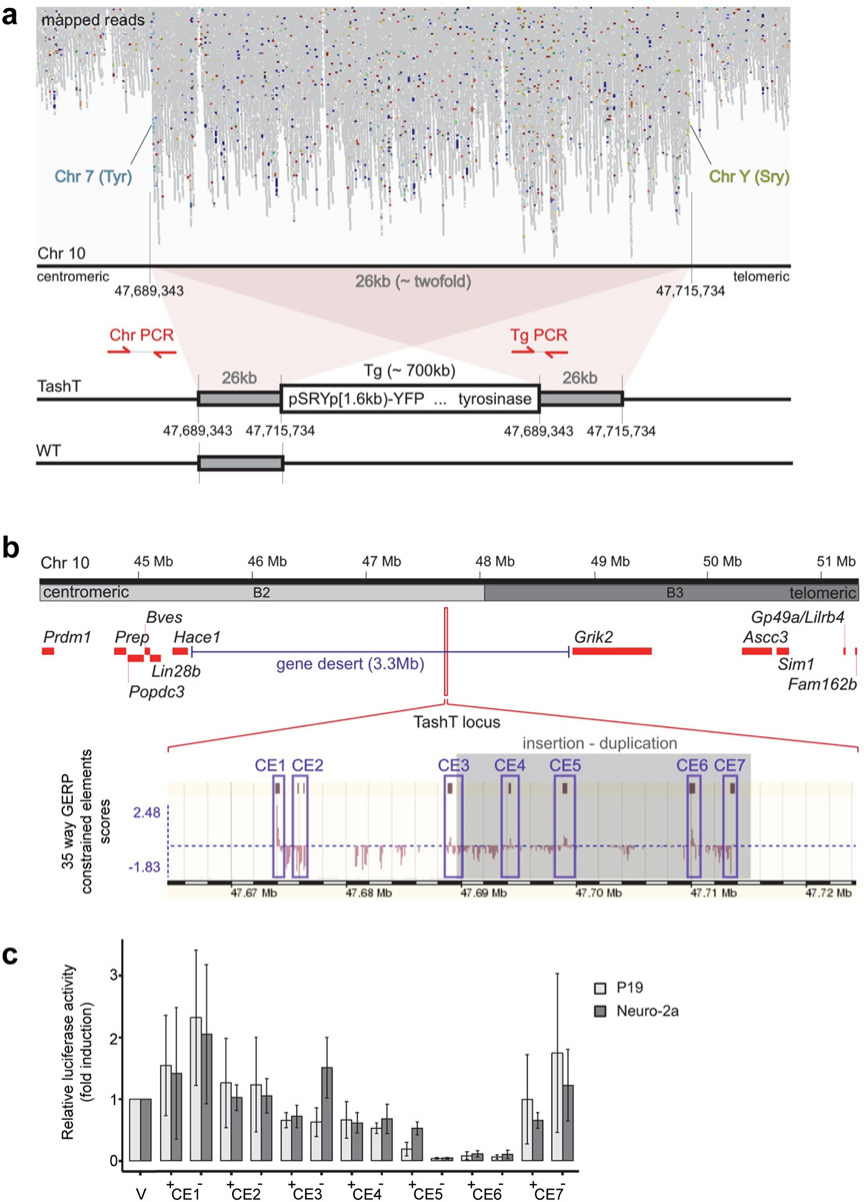
The TashT transgene insertion site is enriched in highly conserved regions possessing silencer activity. **(a)** Mapping of TashT^Tg/Tg^ genome sequencing reads on mouse chromosome 10 B2. Note the approximately twofold increase in reads over a 26 kb region and, flanking it, translocation-like events from chromosome 10 to chromosomes 7 (*Tyrosinase* gene locus) and Y (*Sry* gene locus), in light blue and light green respectively. A schematic representation of the TashT transgene insertion site as deduced from the mapping results is shown at the bottom. Transgenic sequences are estimated to be ~700 kb in length and surrounded by a 26 kb duplication. The position of genotyping PCR primers used in S7b Fig is indicated. **(b)** Map of the chromosome 10 B2–B3 region showing insertion of the TashT transgenes within a 3.3 Mb gene desert. The relative position of protein-coding genes around the locus is indicated by red rectangles. As indicated at the bottom with the GERP (Genomic Evolutionary Rate Profiling) conservation scores for 35 eutherian mammals (taken from www.ensembl.org using the NCBIm37 assembly), this region is enriched in constrained elements. Blue boxes delineate the ~1 kb sub-regions containing the constrained elements that were assessed for transcriptional activity (CE1 to CE7). **(c)** Evaluation of transcriptional activity for the 7 cloned regions in murine neuroblastoma (Neuro-2a) and embryocarcinoma (P19) cell lines. Luciferase assays were performed with reporter constructs driven by the cloned regions (CE1 to CE7) upstream of a minimal TK promoter. Luciferase activity is reported in fold induction relative to the empty vector (V) which is only driven by the TK minimal promoter. +/- symbols indicate sense and antisense orientation of the cloned fragments in relation to the reporter gene.

Breeding of the TashT line revealed systematic co-segregation of pigmentation with YFP fluorescence, meaning co-integration of both transgenes into a single autosomal locus which is frequent when an equimolar mixture of each transgene is micro-injected [15]. FISH analysis first allowed a rough estimate of the localization of the transgene insertion site on chromosome 10 at bands B2–B3 (S7a Fig). To obtain a more precise localization, we sequenced the whole genome of a TashT^Tg/Tg^ mouse. Mapping of high-throughput paired sequencing reads allowed us to localize the transgenic insertion around the middle of a 3.3Mb gene desert between *Hace1* and *Grik2* (Fig. 3a,b). Twice as many reads were observed in a 26kb non-coding region of chromosome 10B2, indicating a duplication. Flanking this duplicated region were paired reads with one end mapping to chromosome 10 and the other end mapping to sequences corresponding to either one or the other transgene (Fig. 3a). A schematic representation of the inferred organization of the TashT transgene insertion site is shown at the bottom of Fig. 3a. The number of transgene copies was estimated from the mapping data and the total size of the insertion calculated to be about 700kb.

The TashT locus contains several blocks of evolutionary conserved non-coding sequences (Fig. 3b). To evaluate their regulatory potential, we cloned seven ~1kb fragments containing most of these constrained elements (CE)—named CE1 to CE7—in a luciferase expression vector bearing a minimal thymidine kinase promoter. Transcriptional activity was then assessed in various cell lines (Neuro-2a, P19, Cos7 and NIH 3T3) via luciferase assays (Figs. 3c and S9). Overall, this analysis revealed very strong repression activity in a cell type-independent manner for two of the cloned regions (CE5 and 6) (Figs. 3c and S9). These luciferase assays thus suggest that the TashT transgenic insertion has disrupted at least one important long-range regulatory element that normally represses expression of a surrounding gene. However, expression of the two most proximal neighboring genes on each side of the gene desert (*Lin28b* and *Hace1* as well as *Grik2* and *Ascc3*) (Fig. 3b) was found to be similar in TashT^Tg/Tg^ and control G4-GFP e12.5 eNCC recovered by FACS (S8 Fig).

### The transcriptional signature of TashT^Tg/Tg^ eNCC

To cast a wider net and detect transcript variation in an unbiased manner, we sequenced the rRNA-depleted transcriptome of FACS-recovered eNCC from anterior intestinal tissues of stage-matched control (G4-GFP) and TashT^Tg/Tg^ e12.5 embryos. This stage was chosen because it combines ease of intestine dissection with clear presence of the eNCC colonization defect in TashT^Tg/Tg^ tissues. It is also important to note that this analysis was restricted to anterior intestinal tissues only (prospective oesophagus, stomach and small intestine) because endogenous YFP labelling in the TashT line is strictly specific to eNCC in these regions (S1b Fig). As mentioned above (see Fig. 2a), the TashT line exhibit ectopic YFP fluorescence in non-NCC derivatives in more posterior regions (prospective cecum and colon) and, therefore, these regions were excluded from both control and mutant cell preparations.

Analysis of RNAseq data revealed that over 1200 coding and non-coding genes are differentially expressed in a significant manner (≥2-fold and *p* <0.001) in TashT^Tg/Tg^ eNCC, among which upregulated and downregulated genes are equally represented (S1 Dataset and S10c Fig). The most deregulated genes (≥4-fold) are listed in Table 1 and this shorter list now indicates a strong enrichment for upregulated genes in TashT^Tg/Tg^ eNCC (41 downregulated vs 188 upregulated). Each of these 229 genes was manually assigned to a category based on function and/or localization of their gene product. Assembling these categories in “super-categories” reveals that TashT affected genes are mostly involved in the control of cell signaling (categories: Ligand-receptor and Signal transduction) and gene expression (category: Transcription factor) as well as in the composition of, and interaction with, the cell microenvironment (categories: Extracellular matrix, Cell adhesion as well as Channel and transmembrane transport). Especially notable examples within each of these super-categories include, respectively, genes encoding Gdnf and Edn3 ligands, many Hox transcription factors as well as various Collagen members. Another category worth mentioning is the Metabolic pathway which notably contains many players of retinoid signaling (Aldh1a1, Aldh1a2, Aldh1a7 and Rdh10). We verified the expression level of selected genes by semi-quantitative RT-PCR. Our selection criteria included genes known as playing a major role in HSCR (Table 2 and S10a Fig) as well as genes located on a sex chromosome whose change in expression could explain the observed male bias (i.e. upregulated on chromosome Y or downregulated on chromosome X) (Table 3 and S10b Fig). All genes tested followed the trend set by the RNAseq data.

**Table 1:** Most significantly deregulated genes in e12.5 TashT eNCC.

Category	Gene names (fold change relative to control)
**Ligand-receptor**	Angptl1 (+5.4), Angptl6 (+5.8), Apln (+4.3), Bmp3 (+4.6), Bmp5 (+6.6), Cckar (+13.0), Chrm2 (+4.2), Clcf1 (+5.2), Crtam (+12.5), Dpp4 (+5.6), Edn3 (+4.8), Fgfr2 (+5.1), Fst (+4.4), Gdnf (+6.3), Gpbar1 (+5.8), Gpr20 (+6.6), Gpr50 (+9.3), Gpr97 (+4.2), Gpr116 (+4.2), Hhip (+4.1), Ifitm1 (+6.1), Il7r (+15.6), Il13ra1 (+4.6), Il17re (+7.1), Il33 (+7.8), Metrnl (+4.9), Ntsr1 (+4.3), Robo4 (+5.3), Rspo1 (+4.1), Rspo3 (+4.2), Sbspon (+8.0), Tacr1 (+4.4), Tnfrsf11b (+8.1), Wnt5a (+4.5), Cartpt (-4.8), Cck (-5.0), Gcg (-13.3), Igsf21 (-4.9), Lrrc4c (-4.7), Npy (-8.7), Prok2* (-2099.7)
**Signal transduction**	Arhgap6 (+4.5), Dock9 (+5.6), Fhl2 (+4.3), Gimap4 (+5.3), Gimap5 (+4.3), Gimap6 (+4.3), Irgm2 (+4.4), Mrvi1 (+5.5), Mx2 (+4.1), Nos2 (+5.5), Otogl (+4.5), Pcp4l1 (+5.9), Pik3r6 (+4.4), Ppef2 (+10.0), Psd (+4.2), Rasgrp3 (+4.4), Samsn1 (+4.7), Sh3rf2 (+7.0), Traf5 (+4.4), Upk1b (+6.0)
**Extracellular matrix**	Adamdec1 (+22.0), Adamts8 (+10.3), Col6a1 (+4.0), Col6a4 (+9.5), Col15a1 (+6.1), Col23a1 (+5.0), Col24a1 (+4.5), Crispld2 (+4.4), Emilin3 (+4.4), Epyc (+6.8), Hmcn2 (+5.2), Hpse2 (+5.0), Lum (+6.1), Mgp (+5.9), Smoc1 (+5.9), Thsd4 (+4.6), Tll1 (+4.2), Tnxb (+9.1)
**Cell adhesion**	Cldn11 (+4.1), Cldn15 (+5.1), Clec1a (+4.1), Hapln3 (+4.1), Iqgap2 (+4.1), Plekhh2 (+4.3), Sdk1 (+5.3), Thbs4 (+5.3), Habp2 (-4.0)
**Metabolic pathway**	Aldh1a1 (+16.1), Aldh1a2 (+7.1), Aldh1a7 (+11.6), Arsi (+4.5), Btn2a2 (+4.9), Ch25h (+6.9), Chst15 (+4.4), Ddo (+11.3), Gbgt1 (+8.7), Ggt5 (+5.4), Got1l1 (+6.0), Hpgd (+4.3), Mgll (+5.9), Ptgs2 (+4.4), Pygl (+4.7), Rdh10 (+4.8), Saa1 (+8.2), Saa2 (+7.9), Tdo2 (+4.9), Tyr (+4.6), B3gat1 (-5.1), Dbh (-4.1), Gulo (-5.0), Hs3st6 (-4.7), Hsd3b6 (-5.3)
**Transcription factor**	Bcl6b (+4.1), Bhlhe40 (+4.0), Foxf1 (+5.3), Foxf2 (+6.5), Foxl1 (+5.5), Gli1 (+5.9), Hand1 (+5.0), Hoxa7 (+4.0), Hoxc6 (+10.2), Hoxc8 (+17.7), Hoxc9 (+5.0), Hoxd8 (+4.6), Nkx2–3 (+5.5), Sox7 (+4.1), Tcf15 (+4.4), Tcf21 (+4.0), Zfp366 (+4.5), Ankrd1 (-4.5), Dmrt3 (-5.9), Hoxa10 (-51.3), Hoxa11* (-728.6), Hoxd9 (-9.1), Hoxd10* (-324.5), Hoxd11* (-943.6), Isx (-4.9), Lmo1 (-4.2), Onecut3 (-6.2), Pou3f3 (-40.7), Pou4f2 (-10.4), Sox8 (-4.1)
**Channel and trans-membrane transport**	Ano1 (+4.4), Atp13a4 (+9.4), Cacnb2 (+4.9), Clca5 (+5.1), Kcnd3 (+5.9), Kcng1 (+9.0), Kcnh1 (+6.0), Kcnip1 (+4.6), Kcnmb2 (+8.0), Slc4a10 (+24.9), Trpc4 (+4.6), Cacna2d3 (-4.1), Chrna4 (-4.5), Slc6a2 (-5.2), Slc38a5 (-13.7), Tmem27* (-32.4)
**Muscle-associated**	Acta2 (+6.5), Actg2 (+6.6), Cnn1 (+6.7), Lmod1 (+5.2), Myh11 (+5.9), Mylk (+6.6), Myo1h (+6.9), Myocd (+6.9), Myom1 (+4.6), Pamr1 (+7.1), Sntg2 (+7.2), Synpo2 (+6.8), Mlip(-4.8), Myl1 (-4.4)
**Miscellaneous**	Acap1 (+5.3), Asb2 (+6.6), Colec10 (+8.3), Dnahc6 (+4.7), Erp27 (+7.9), Esm1 (+5.9), Eva1a (+4.2), Exoc3l (+4.7), Fabp4 (+8.1), Gpihbp1 (+9.5), Lsp1 (+4.4), Mir143 (+5.1), Mir145 (+6.7), Prnd (+4.6), Rassf9 (+7.3), Sdpr (+4.6), Sycp2 (+5.0), Upk3b (+5.2), Wdr66 (+5.8), Alas2 (-4.5), Crym (-6.0), Eif4e3 (-4.4), Lin28a (-5.2),
**Uncharacterized (protein coding)**	3425401B19Rik (+14.3), 4930444P10Rik (+8.6), 4932418E24Rik (+10.2), Cped1 (+5.6), Fam65c (+6.0), Fam162b (+5.0), Gm10134 (+7.1), Gm11541 (+5.1), Gm15319 (+5.8), Klhl38 (+6.1), Ssu2 (+4.5), Tmem255a (+5.1), Ushbp1 (+7.2), 3110047P20Rik (-5.3)
**Uncharacterized (ncRNA)**	1700018A04Rik (+6.9), 1700095B22Rik (+11.3), 9330158H04Rik (+5.4), A730056A06Rik (+5.1), F730043M19Rik (+5.2), Fendrr (+9.5), Gm10664 (+4.7), Gm11624 (+11.4), Gm12947 (+11.2), Gm13889 (+6.0), Gm20467 (+7.6), Gm2830 (+4.2), Mir143hg (+5.5), Sec1 (+10.0), 2610017I09Rik (-12.8), A730036I17Rik (-5.1), Gm26748 (-5.2)

Note: Genes included were those whose expression was modulated at least 4-fold (*p* < 0.001). Asterisks indicate genes for which transcript count was null in at least one of the RNA samples, skewing the fold ratio toward exaggerated levels. Negative fold changes are underlined.

**Table 2:** Deregulated genes in e12.5 TashT^Tg/Tg^ eNCC that have been implicated in HSCR and/or ENS formation.

Gene	Function	Fold Change	TashT Mean Raw Count	G4.GFP Mean Raw Count	Chr	Gene Start (bp)	Gene End (bp)
Aldh1a1	retinoic acid metabolism	16,09	8 108	540	19	20 601 961	20 643 462
Aldh1a2	retinoic acid metabolism	7,14	12 001	1 824	9	71 215 789	71 296 243
Ece1	endothelin processing	2,30	8 084	3 758	4	137 862 237	137 965 229
Edn3	secreted ligand for Ednrb	4,80	1 875	432	2	174 760 619	174 784 042
Ednrb	Edn3 receptor	-2,80	20 420	57 755	14	103 814 625	103 844 173
Gdnf	secreted ligand for Ret	6,27	2 477	428	15	7 811 011	7 837 575
Hand2	transcription factor	-2,07	4 355	9 135	8	57 320 983	57 324 517
Hlx	transcription factor	3,57	2 736	841	1	184 727 140	184 732 619
Ntf3	neurotrophic ligand	3,52	494	153	6	126 101 413	126 166 744
Phox2b	transcription factor	-3,31	4 950	16 278	5	67 094 397	67 099 249
Ret	Gdnf receptor	-3,78	9 204	34 338	6	118 151 748	118 197 744
Sox10	transcription factor	-2,97	2 229	6 527	15	79 154 913	79 164 490

Note: Genes included were those whose expression was modulated at least 2-fold (*p* < 0.001).

**Table 3:** Downregulated X-linked genes in e12.5 TashT^**Tg/Tg**^ eNCC.

Gene	Function	Fold Change	TashT Mean Raw Count	G4.GFP Mean Raw Count	Gene Start (bp)	Gene End (bp)
Clcn5	ion channel	-2,14	4 078	8 936	7 153 810	7 319 358
Syp	vesicular trafficking	-2,89	382	1 125	7 638 471	7 653 256
Slc38a5	amino acid transport	-13,72	14	202	8 271 133	8 280 179
Lancl3	signal transduction	-2,91	117	341	9 199 902	9 268 085
Tspan7	signal transduction	-2,13	1 361	2 980	10 485 158	10 596 605
Gm5124	pseudogene	-2,17	117	261	21 360 865	21 364 622
Zcchc12	transcription factor	-2,32	495	1 165	36 195 904	36 199 158
Arhgap36	Rho GTPase activating protein	-2,15	151	335	49 463 945	49 500 244
Cxx1c	uncharacterized (protein-coding)	-2,18	258	573	53 607 922	53 609 132
Fgf13	growth factor	-2,68	690	1 871	59 062 145	59 568 071
Slitrk2	signal transduction	-2,67	968	2 623	66 649 318	66 661 393
Rab39b	vesicular trafficking	-2,60	402	1 065	75 572 046	75 578 231
Nlgn3	neuronal cell-cell interaction	-2,46	203	509	101 299 168	101 325 963
Nap1l2	chromatin regulation	-2,34	279	670	103 184 176	103 186 640
5330434G04Rik	uncharacterized (lincRNA)	-2,02	347	707	105 348 282	105 391 776
Bex1	signal transduction	-2,16	764	1 693	136 213 972	136 215 513
Plp1	major myelin protein	-2,43	1 289	3 194	136 822 671	136 839 733
Rab9b	vesicular trafficking	-2,95	78	232	136 858 147	136 868 755
Dcx	microtubule-associated	-3,17	1 103	3 517	143 855 842	143 933 311
Alas2	heme biosynthesis	-4,46	33	150	150 547 375	150 570 638
Map7d2	microtubule-associated	-2,47	158	398	159 414 572	159 498 757
Tmem27	amino acid transport	-32,40	1	39	164 088 830	164 118 860

Note: Genes are ordered by chromosomal location and consist of those whose expression was modulated at least 2-fold (*p* < 0.001). No Y-linked genes were found to be significantly upregulated.

Given that intra-chromosomal regulatory chromatin contacts are much more prevalent than inter-chromosomal ones [26], the selection criteria for identification of the TashT causative gene was its location relative to the repressive elements disrupted by the transgene insertion. We therefore focused on the handful of upregulated genes located on chromosome 10 (Table 4) in our search for a gene directly regulated by the conserved elements. The closest candidate, *Fam162b*, is ~3.6 Mb telomeric to the conserved elements (Fig. 3b) and its overexpression in TashT^Tg/Tg^ eNCC was validated via semi-quantitative RT-PCR (S11a Fig). Note that *Fam162b* mRNA can also be detected in eNCC FACS-sorted from control embryos, indicating its low level expression in normal eNCC populations (see S1 Dataset). Analyses of *Fam162b* open-reading frame sequences using *ExPASy* (www.expasy.org) and Uniprot (www.uniprot.org) resources suggest that this uncharacterized gene encodes a single pass transmembrane protein localized to mitochondria.

**Table 4:** Chromosome 10 most significantly deregulated genes in e12.5 TashT^Tg/Tg^ eNCC.

Gene	Function	Fold Change	TashT MeanRaw Count	G4.GFP MeanRaw Count	Gene Start (bp)	Gene End (bp)
Tcf21	transcription factor	4,01	4 671	1 269	22 817 279	22 820 128
Rspo3	activator of Wnt pathway	4,24	2 542	649	29 453 109	29 535 867
Ddo	oxidative deamination of aspartate	11,29	77	7	40 630 011	40 649 931
**Fam162b**	**uncharacterized (protein coding)**	4,99	391	85	51 585 420	51 590 480
Ggt5	converts leukotriene C4 to D4	5,40	231	46	75 589 381	75 616 968
Col6a1	extracellular matrix, collagen	4,02	10 966	3 004	76 708 792	76 726 168
Onecut3	transcription factor	-6,24	11	68	80 494 835	80 517 276
Lum	extracellular matrix, proteoglycan	6,07	5 146	943	97 565 501	97 572 703
Epyc	extracellular matrix, proteoglycan	6,81	401	65	97 644 068	97 682 454
Rassf9	vesicular trafficking	7,29	471	72	102 512 222	102 546 560
Otogl	inner ear mechanotransduction	4,50	262	63	107 762 223	107 912 134
Gli1	transcription factor	5,86	3 209	585	127 329 889	127 341 589

Note: Genes included were those whose expression was modulated at least 4-fold (*p* < 0.001).

### Overexpression of Fam162b may explain the TashT ENS defect

**Fig 4:**
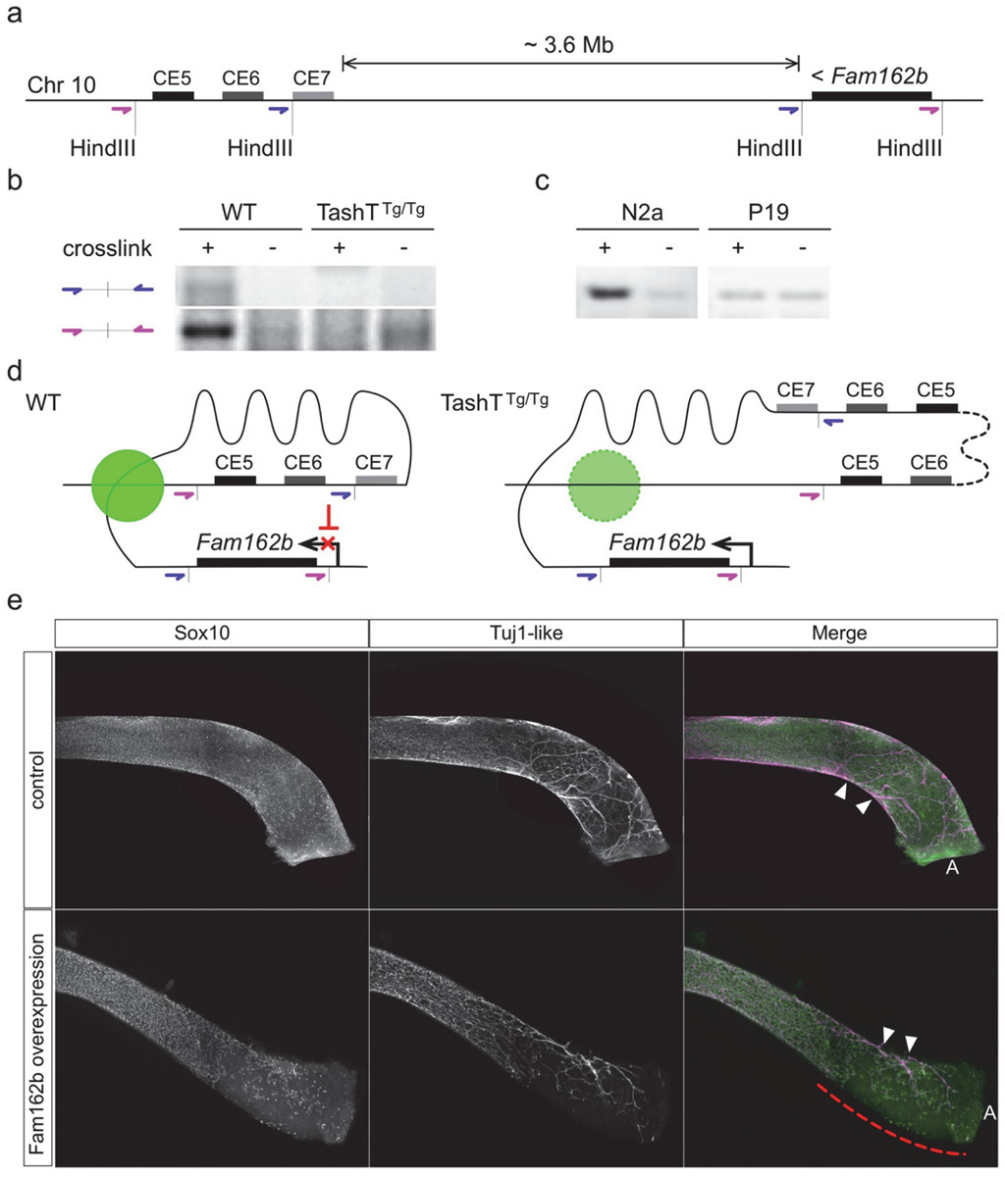
Transgene insertion-mediated relief of *Fam162b* repression in NCC contributes to the TashT ENS defect. **(a)** Diagram depicting the relative position of *Fam162b* and the conserved silencer elements (CE5 and CE6) of the TashT insertion site on chromosome 10, and placement of the HindIII restriction sites and upstream oligonucleotide pairs (in purple and blue) used for chromosome conformation capture (3C). **(b-c)** 3C PCR amplification results obtained from wildtype FVB/n and TashT^Tg/Tg^ e12.5 whole embryonic intestines (**b**; about 1x10^6^ cells per library) as well as from Neuro-2a and P19 cell lines (**c**; 1x10^8^ cells per library). Non-crosslinked negative controls were included for each library. **(d)** Model of chromatin interaction in NCC between *Fam162b* and the conserved silencer elements. The green circle represents a putative protein complex facilitating this specific interaction. In TashT cells, the large transgene insertion (dotted line) along with the short chromosome 10 duplication disrupts normal chromatin looping conformation and thereby leads to relief of *Fam162b* repression. **(e)** Validation of *Fam162b* as being involved in TashT ^Tg/Tg^ pathogenesis via transient transgenesis in e15.5 embryos. *Fam162b* coding sequences were overexpressed in NCC under the control of a previously described *Sox10* enhancer (U3) and the impact on ENS development was assessed using Sox10 and βIII-Tubulin as markers. The dashed red line indicates the incomplete eNCC colonization in the distal colon of a female *Fam162b* overexpressing embryo. Arrowheads point to extrinsic innervation entry sites. A, anus.

We subsequently verified that the conserved silencer elements near the transgene insertion site normally interact with the *Fam162b* locus using chromosome conformation capture (3C) techniques [27]. These analyses first revealed that such interaction can be detected in wild-type whole embryonic intestines using different primer pairs (Fig. 4a,b). We also found that this interaction is cell type-specific as it is detected in NCC-derived Neuro-2a cells but not in undifferentiated P19 cells (Fig. 4c), the same murine embryonic cell lines used in our luciferase assays (Fig. 3c). Importantly, we further found that this interaction is lost in TashT^Tg/Tg^ embryonic gut tissues (Fig. 4b). Therefore, these results strongly suggest that the TashT transgenic insertion disrupts intra-chromosomal contacts that normally repress *Fam162b* expression in NCC (Fig. 4d).

To independently validate the candidacy of *Fam162b* as being involved in the TashT ENS defect, we generated transgenic e15.5 embryos specifically overexpressing *Fam162b* in NCC and analyzed the impact on ENS formation using specific markers. The transgenic construct consisted of a bicistronic cassette containing *Fam162b* and *eGFP* coding sequences driven by a previously described *Sox10* enhancer (U3, also known as MCS4) fused to the *Hsp68* minimal promoter [28,29]. Western blotting confirmed that the cloned *Fam162b* sequences express a protein of expected size (S11b Fig). Using fluorescence from eGFP as a surrogate marker for transgene expression, we obtained a total of three *Fam162b* transgenic embryos (all females) exhibiting variable levels of transgene expression. In contrast to littermate controls for which bowel tissues were fully colonized, all three transgenic embryos displayed incomplete colonization of the distal hindgut by vagal-derived eNCC and, as a result, this region was found to only contain scattered Sox10-positive eNCC of presumably sacral origin in a way similar to what is observed in TashT^Tg/Tg^ tissues (compare Fig. 4e with Fig. 2a). In addition to having used a different genetic background for these experiments (B6C3), we believe that the fact that all *Fam162b* overexpressers were female likely explains, at least in part, the more modest effect observed in comparison to TashT^Tg/Tg^ embryos. Although we cannot currently exclude the possibility that other gene(s) might also be primary target(s) of the TashT mutation and might thus also contribute to the TashT^Tg/Tg^ phenotype, these transgenesis data support a role for *Fam162b* overexpression in TashT^Tg/Tg^ pathogenesis.

## Discussion

We have identified and characterized a novel mouse model for aganglionic megacolon, called TashT, allowing us to describe the eNCC transcriptome during development and to propose two novel genetic loci implicated in the pathogenesis of megacolon—one that is protein coding, the other involving evolutionary conserved non-coding sequences—as well as an ultra-long-range interaction between these loci. In addition, this mouse model allows us to speculate on the mechanistic nature of the hitherto unexplained male bias of the megacolon phenotype observed in humans, as well as its variable penetrance.

### The transcriptional signature of TashT^Tg/Tg^ eNCC: deregulation of Gdnf/Ret and Edn3/Ednrb signaling

To the best of our knowledge, this study is the first to report a transcriptome analysis of sorted eNCC. Previous screens for genes expressed in eNCC were performed on whole embryonic intestines using DNA microarrays and based on the differential expression between normal and *Ret*-null aneural tissues [30,31]. In addition to analyzing eNCC directly, we took full advantage of the RNAseq technology and included non-coding RNA in our analyses. This resulted in a much more extensive list of genes known to be expressed in eNCC, from a few hundred to several thousand (S1 and S2 Datasets).

Importantly, the transcriptional signature of TashT^Tg/Tg^ eNCC not only highlighted *Fam162b* as a potential causative gene, but also provided mechanistic insights into the identified cell migration defect. In this regard, it is noteworthy that several modulated transcripts in TashT^Tg/Tg^ eNCC encode components of the extracellular matrix (ECM) (Table 1 and S10c Fig). NCC have been suggested to modify their extracellular environment during, or perhaps as a requirement for, migration [32,33]. One possibility is thus that the modulated ECM in TashT^Tg/Tg^ embryonic intestines is less permissive to cell migration [34,35]. Another, not mutually exclusive possibility is that the reduced sensitivity of TashT^Tg/Tg^ eNCC to growth factors/chemoattractants normally present in the intestinal ECM underlies the migration defect. In agreement with this, we have found that TashT^Tg/Tg^ eNCC have lost their ability to respond to exogenous GDNF and EDN3 in explant assays, with eNCC appearing unable to distinguish between bowel tissue and collagen gel (Fig. 2d). This latter outcome is most likely due to the surprising robust overexpression of both *Gdnf* and *Edn3* by TashT^Tg/Tg^ eNCC (Table 2 and S10a Fig). Indeed, given that Gdnf and Edn3 are both normally heavily secreted from the surrounding mesenchyme, additional oversecretion from eNCC is expected to disturb the dosage of ligands these cells normally encounter and/or to disrupt any gradient that might be present. Our RNAseq data also suggest that the lack of responsiveness to GDNF and EDN3 might be due to an overabundance-induced negative feedback on their cognate receptor. This hypothesis is supported by the fact that expression of both *Ret* and *Ednrb* is reduced in TashT^Tg/Tg^ eNCC (Table 2 and S10a Fig). Interestingly, in the case of Gdnf/Ret signaling, this hypothesis is further supported by the observed overexpression of *Lrig1* and *Lrig3* in TashT^Tg/Tg^ eNCC (S1 Dataset). These genes encode functionally-redundant transmembrane proteins [36] which, as specifically demonstrated for Lrig1 in neuronal cells, can be induced by Gdnf at the transcriptional level and then physically interact with Ret in order to reduce Gdnf binding and tyrosine kinase activity [37]. Regardless of the exact underlying mechanism, a lack of responsiveness to EDN3 might well be responsible for the slower migration of TashT^Tg/Tg^ eNCC (Figs2b-c and S4), as inhibition of Ednrb signaling has been recently shown to primarily affect the speed of eNCC migration [38].

### Long-range repression of Fam162b expression

Most of the known and characterized long-range acting regulatory elements do not interact with their nearest promoter but bypass several intervening genes in order to reach their target promoter [39]. Long-range enhancer-promoter interactions are also thought to be more commonly involved in the regulation of tissue-specific genes [40]. Moreover, most studies of intra-chromosomal long-range interactions involve loci up to several hundred kb away from each other, though ultra-long-range events (several Mb) between enhancer and promoter are not uncommon [27,40,41]. Our 3C data are in accordance with these observations and indicate that the interaction between the conserved elements near the TashT transgene insertion site and the *Fam162b* gene ~3.6 Mb away (Fig. 4b) falls in the ultra-long-range category. Little is known of the spatiotemporal pattern of *Fam162b* expression. The evidence to date is in agreement with an expression in neural derivatives: it is weakly expressed in the mouse olfactory bulb (Allen Brain Atlas, RP_051012_01_G06) and expressed in the frontonasal prominence of mouse embryos, proximal to the oral cavity [42]—a tissue heavily populated by cranial NCC. These observations are consistent with the idea that *Fam162b* is poised for active transcription in neural and/or neural crest cells but kept in check through a repressive mechanism.

Using luciferase assays, we demonstrated that a subset of the highly conserved elements near the TashT transgene insertion site has a robust negative regulatory function on transcription in a cell type-independent manner (Fig. 3c and S9 Fig). Given the forced juxtaposition of regulatory elements with the proximal promoter in such assays, the absence of a cell type-specific activity in our analysis thus points to a chromatin conformation-dependent mechanism conferring specificity *in vivo*. As supported by our 3C data (Fig. 4c), we suggest that an ultra-long-range chromatin loop maintains *Fam162b* expression at a basal level in a subset of neural-derived cells, including eNCC. Further investigations into the regulation of *Fam162b* expression will be necessary to confirm and expand this hypothesis. The biological function of the *Fam162b* gene product is also currently unknown. Characterization of this function will clearly be facilitated by the wealth of information obtained from the RNAseq data as well as by the observed cell migration defect.

### The source of the male bias

Robust correlation between extent of aganglionosis and expressivity of the megacolon phenotype in TashT^Tg/Tg^ animals allowed us to identify the minimal distance of myenteric innervation necessary for successful movement of luminal content across the colon in FVB/n mice. This critical region (~80% of colon length; Fig. 1e) represents a threshold level beneath which intestinal blockage occurs systematically, and in a sex-independent manner. The fact that the mean length of the ganglionated region of TashT^Tg/Tg^ males ends in this critical region while females typically show a more extensive ENS explains the apparent contradiction between the male bias in megacolon expressivity and the near complete penetrance of distal aganglionosis in both sexes. A common defect of eNCC colonization, slightly exaggerated in males, is thus the source of the observed sex bias of the megacolon phenotype in TashT^Tg/Tg^ animals. In this regard, it is noteworthy that a similar link between extent of aganglionosis and megacolon expressivity has been previously described in mice bearing the *Ednrb* ^s-l^ allele [43] as well as in *Ret* ^+/-^::*Ednrb* ^s/s^ compound mutants [14]. Interestingly, although only a very modest male sex bias in megacolon expressivity was reported in this latter case (~1.5:1), the correlations made with extent of aganglionosis are in agreement with the threshold level revealed by our study. Indeed, full penetrance of megacolon in *Ret* ^+/-^::*Ednrb* ^s/s^ males was correlated with a mean length of the ganglionated region clearly beneath the threshold (59% of colon length) whereas partial penetrance of megacolon in *Ret* ^+/-^::*Ednrb* ^s/s^ females was correlated with a mean length of the ganglionated region much closer to the threshold (72%) [14]. However, as evidenced by the fact that *Sox10Dom* mutants on a C57BL/6J—C3HeB/FeJ mixed background display a shorter aganglionic zone leading to megacolon (~10%), it should also be noted that position of the threshold level may vary as a function of the genetic background [44].

The aganglionic megacolon of TashT^Tg/Tg^ animals share striking similarities with both the variable penetrance and male sex bias of short-segment HSCR, the most common form of the disease (~80% of cases). The threshold level identified with the TashT line is also in accordance with the fact that virtually no sex bias is observed in long-segment HSCR. Our analysis of the TashT line thus provides useful insights into the ontogeny of aganglionic colon and the origin of the sex bias, and shows that, though perhaps suffering from chronic constipation, TashT^Tg/Tg^ mice are nevertheless able to pass intestinal material when more than 4/5 of their colon is innervated.

Apart from *Ret* ^+/-^::*Ednrb* ^s/s^ compound mutants, it is interesting to note that a male bias in the extent of aganglionosis—but not megacolon expressivity—has also been reported in other mouse and/or rat models and in each case implicated a mutation in either *Ret* or *Ednrb* [13,45,46]. Taken together with our data showing deregulated Ret and Ednrb signaling in TashT^Tg/Tg^ animals as well as with the previous description of a *RET* non-coding mutation that is twice as frequently transmitted in boys than in girls [47], these observations strongly suggest that both pathways are involved in the regulation of expression of a still undefined sex chromosome-linked gene with critical function in the developing ENS. We reasoned that exaggerated defects in males could arise from overexpression of male-specific genetic material (upregulated genes on Y chromosome) or from a deficiency in the expression of genes present as single alleles (downregulated genes on X chromosome). Another interpretation of this second possibility is that females are protected through biallelic expression of some X chromosome genes, provided they escape X-inactivation [48–51]. Analysis of our transcriptome dataset revealed that expression of Y-linked genes in eNCC is limited to a group of four genes (*Kdm5d*, *Eif2s3y*, *Uty* and *Ddx3y*) (S2 Dataset). Of these clustered genes—also known to be expressed in the developing brain [52,53]–, *Ddx3y* is the only one that approaches significant upregulation in TashT^Tg/Tg^ eNCC (1.5-fold; edgeR *p* = 0.0011; DESeq *p* = 0.0055). In marked contrast, multiple X-linked genes were found to be significantly downregulated in TashT^Tg/Tg^ eNCC, including *Dcx*—a previously suggested potential HSCR susceptibility locus (Table 3) [30]. As X-inactivation escapees tend to be found in clusters [48], other interesting candidates include a group of genes—in the vicinity of *Dcx*—that contains *Bex1* and the *Plp1-Rab9b* gene pair (Table 3). It is noteworthy that the candidacy of these genes is also supported by human cases with Xq22 microdeletions encompassing them. Indeed, while such cases are assumed to be embryonic lethal in males, female patients suffer from Pelizaeus-Merzbacher-like disease with symptoms of gastrointestinal motility problems including constipation [54].

## Materials and Methods

### Animals

Work with mice was performed in accordance with the guidelines of the Canadian Council on Animal Care (CCAC) and approved by the relevant institutional committee (*Comité institutionnel de protection des animaux*; CIPA reference #650) of University of Quebec at Montreal (UQAM). Mice were euthanized by gradual-fill carbon dioxide (CO_2_) gas preceded by isoflurane anesthesia. TashT transgenic mice and *Fam162b* transgenic embryos were generated via standard pronuclear microinjection [55], using embryos derived from FVB/n albino and B6C3 mice, respectively. For the TashT line, two transgenes were co-injected at equimolar ratio: a *Tyrosinase* minigene to allow visual identification of transgenic animals via rescue of pigmentation [15] and a pSRYp[1.6kb]-YFP construct that provides fluorescent marking of migrating NCC in the developing embryo [16]. The previously described Gata4p[5kb]-GFP line (G4-GFP) was used as wild-type control [17]. Mice were mated overnight and noon on the day a vaginal plug was observed was designated as embryonic day (e) 0.5. For *Fam162b* transient transgenics, a transgene carrying the PCR-amplified *Fam162b* open reading frame under the control of the *Hsp68* minimal promoter [56] and a NCC-specific *Sox10* enhancer (U3, also known as MCS4) [28,29] was used. In order to provide a positive control for transgene expression, an IRES-GFP cassette (pIRES2-EGFP, Clontech) was also included immediately downstream of the *Fam162b* ORF, creating a bicistronic message. Fifteen days after microinjection, foster mothers were sacrificed, embryos were collected, individually analyzed for GFP and immunostained for Sox10 and βIII-Tubulin (see immunofluorescence section below).

### Fluorescence in situ hybridization (FISH) of mouse chromosomes

Antisense and sense digoxigenin-labelled RNA probes were synthesized using a DIG transcription kit (Roche). Mouse spleen lymphocytes were collected and metaphase slides prepared using standard cytogenetic protocols [57,58]. Slides were aged at room temperature for 7 days, and then GTG banded, again using standard protocols [55]. Slides were scanned at 100X using a Nikon Eclipse E800 microscope, and representative metaphases were photographed at 1000X using a Nikon DXM1200 digital camera and SimplePCI software. Slides were de-stained using CitriSolv (Fisher) for 10–15 minutes, fixed for 1 minute in a 1% paraformaldehyde (PFA) solution in 2X SSC then dehydrated in graded ethanol washes and stored for future use in 100% ethanol. Slides were air dried then incubated in a denaturation solution for 5 minutes at 73°C, then again dehydrated in graded ethanol washes and stored in 100% ethanol. Probe was generated using a digoxigenin-labeled *Tyr* minigene DNA fragment [57]. Just prior to adding the denatured FISH probe, slides were air dried. Five to 10 μl of denatured probe was added per slide, which was then incubated overnight at 37° under a coverslip in a humidified dark atmosphere. Detection was performed using a rhodamine-conjugated anti-DIG antibody (Roche), following the manufacturer’s instructions. Slides were counterstained with DAPI (Sigma) and antifade (P-phenylenediamine; Sigma). Previously photographed G-banded metaphases were re-located using epifluorescence, rephotographed, and compared to FISH images.

### Fluorescence-activated cell sorting (FACS)

Intestines were dissected from e12.5 G4-GFP and TashT embryos without their posterior end (prospective cecum and colon) because, in contrast to more anterior regions in which only eNCC are fluorescently-labelled (see S1b Fig), TashT intestines also contain high amounts of YFP-positive mesenchymal cells that are not derived from NCC in these regions (see Figs. 2a and S4). A MoFlo XDP (Beckman Coulter) cell sorter was used to collect GFP- or YFP-positive single viable cells from the remaining dissociated intestinal tissue. Dissociation was carried out at 37°C in EMEM containing collagenase (0.4 mg/ml; Sigma C2674), dispase II (1.3 mg/ml; Life Technologies 17105–041) and DNAse I (0.5 mg/ml; Sigma DN25).

### High throughput genome and transcriptome sequencing

Whole genome and transcriptome library generation and sequencing was performed by McGill University and Génome Québec Innovation Centre using the HiSeq 2000 platform (Illumina). Fifty to 150 million paired-end sequences (100 bp length) were obtained from 300–500 bp library inserts, resulting in an overall 30x coverage for genome reads. Sequences were filtered based on quality and mapped onto the *Mus musculus* reference genome (mm9 for genomic DNA, mm10 for RNA). For the transcriptome, total RNA was rRNA-depleted before making the libraries. Three libraries were generated for each cell population, though only two were of sufficient quality for subsequent bioinformatic analysis. DESeq and edgeR differential gene expression analyses (adjusted p-value < 0.001), as well as a minimum 2-fold expression difference, were taken into account to determine significantly deregulated messages between FACS-recovered eNCC from control (G4-GFP) and TashT^Tg/Tg^ embryos. Gene Ontology analyses were performed using GOToolBox, and at least 2-fold enriched/depleted categories were selected from a hypergeometric test with a Benjamini-Hochberg corrected *p*-value threshold of 0.01 (http://genome.crg.es/GOToolBox).

### Immunohistochemistry (IHC)

Dissected postnatal intestines were fixed in 4% PFA overnight at 4°C and embedded in paraffin. Sections (7 μm) were stained by immunohistochemistry according to standard techniques. Briefly, the paraffin was removed from slide mounted sections by washing with xylene and ethanol, and sections were treated for antigen retrieval with boiling sodium citrate pH 6.0 for 10 min. Slides were blocked for 1 h in blocking solution (1% bovine serum albumin, 1% milk, in Tris-buffered saline pH 7.5), then incubated overnight at 4°C with mouse anti-βIII-Tubulin (Abcam ab78078, 1:200) primary antibody. Following several Tris-buffered saline (TBS) pH7.5 washes, sections were incubated for 2h at room temperature (RT) in alkaline phosphate-conjugated anti-mouse secondary antibody (Abcam ab97043, 1:200). After two washes in TBS pH 7.5 and one at pH 9.5, the staining was revealed with a nitro blue tetrazolium (NBT, 500μg/ml) and 5-bromo-4-chloro-3-indolyl-phosphate (BCIP, 187.5μg/ml) solution (Roche Applied Science) for 10 to 20 minutes. The reaction was stopped with a solution of TBS pH7.5 containing EDTA (20mM) and the slides were mounted with glycerol mounting medium (DAKO).

### Immunofluorescence (IF)

For embryonic tissues, freshly dissected intestines were fixed 1 hour at RT with 4% PFA in PBS. Alternatively, whole embryos were fixed overnight at 4°C and the intestines dissected afterwards. For adult tissues, whole intestines were fixed in 4% PFA overnight at 4°C, cut longitudinally along the mesentery and washed in PBS. The outermost muscle layers were then stripped from the mucosa/submucosa. Fixed tissues were dehydrated in methanol. After rehydration, the intestines were incubated 2 hours at RT in blocking solution (10% fetal bovine serum, 0.1% Triton-X100 in PBS). Tissues were incubated with primary antibodies overnight at 4°C. The antibodies used were: mouse anti-βIII-Tubulin (1:200; Abcam ab78078), rabbit anti-S100β (1:500; Dako Z0311), goat anti-Sox10 (1:100, Santa Cruz Biotech. sc-17342) and rabbit anti-Ki67 (1:1000; Abcam ab15580). Secondary antibodies Alexa Fluor 594- or Alexa Fluor 647-conjugated anti-goat, -mouse or -rabbit (1:500, Jackson Immunoresearch) were incubated for 2 hours at RT and counterstained with DAPI. All antibodies were diluted with blocking solution. For the TUNEL assay, tissues were permeabilized 20 min at 37°C in 0.3% Triton-X100, 0.1% sodium citrate in PBS 1x, then stained in a 1:9 mix of enzyme solution:label solution from the *in situ* cell death detection kit, TMR red (Roche Applied Science 12156792910) 1h at 37°C.

### Acetylcholinesterase staining

The whole colon (from cecum to anus) was dissected from adults and neonates. It was cut longitudinally along the mesentary (adult tissues only), rinsed, pinned flat and fixed in 4% PFA O/N at 4°C. Staining was performed on tissues as previously described [59]. Following the staining procedure, muscle strips were prepared as described above (adult tissues only).

### Ex vivo time-lapse imaging of eNCC

Live imaging of eNCC was performed using a suspended culture technique adapted from Nishiyama et al., 2012 [3]. The abdomen of TashT e11.0 embryos was opened and the surrounding tissues trimmed just enough to expose the developing intestine. The embryo was placed on a small nitrocellulose filter (Millipore GSWP01300) soaked in PBS, and the extra tissues surrounding the intestine were slightly pressed onto the membrane. The PBS was then blotted off before being replaced with DMEM/F12 media (containing 10% FBS and antibiotics). The filter was flipped on top of a DMEM/F12-filled 2 mm-wide trough in a 1% agarose film covering the round glass bottom of a 35 mm culture dish (Greiner Bio One 627860), so that the intestine would float in media without touching either the agarose or the glass. The dish was incubated at 37°C, 5% CO_2_ during 6 hours, while 250μm-thick stacks were acquired with a 10x objective and a Nikon A1R confocal unit. Cell morphology viewed by YFP fluorescence allowed us to label the center of the cell located at the tip of a chain of migrating eNCC at each timeframe. Five to 6 chain tip cells were tracked from at least 3 intestines of each genotype. Speed and directionality were calculated from this dataset, with the orientation of the mesentery as a reference angle. We cannot totally exclude the possibility that more than one cell was included per chain tip measurement as individual eNCC at the wavefront sometimes exchange places with one another by a leapfrogging process [38].

### Ex vivo eNCC migration assays

*Ex vivo* cell migration assays were performed as previously described [23]. Collagen gels containing or not GDNF (10 ng/ml, Cedarlane CLCYT305–2) and/or EDN3 (250 ng/ml, Sigma E9137) were prepared at least 1 hour before use and kept at 37°C in a CO_2_ (5%) incubator. Vibratome transverse sections (200 μm) of embryonic small intestines were put down on the collagen gels for 3 days at 37°C with 5% CO_2_ to let cells migrate into the gel. Bowel sections were removed and cells within the gels were fixed with 4% PFA for 1 hour at RT before being stained with DAPI (Sigma-Aldrich) to detect cell nuclei. All images were taken at 70X magnification with a Leica M205FA fluorescence stereomicroscope as described below.

### Image processing and cell counting

YFP expression in TashT tissues or in migrating cells was visualized using a Leica M205FA fluorescence stereomicroscope. IHC slides were observed using a DM2000 Leica upright microscope. Images were acquired with a Leica DFC495 digital camera and Leica Application Suite (LAS) software (Leica microsystems). IF of stained intestines were examined using an inverted Nikon TI microscope. Images were acquired with a Nikon A1 confocal unit and NIS-Element AR4 software, using standard excitation and emission filters for visualizing DAPI, YFP, Alexa Fluor 594 and 647, as well as spectral imaging coupled with linear unmixing in order to distinguish between YFP and GFP fluorescence. All images were processed with ImageJ software [60]. Image J was also used for cell counting with the *analyze particles* function, or with the *cell counter* manual function.

### Luciferase reporter assays

Constrained elements around the TashT insertion site were identified using Ensembl’s 35 eutherian mammals multiple alignment track (EPO_LOW_COVERAGE) on the NCBIm37 mouse genome version (www.ensembl.org). Seven regions named CE1 to CE7 (ranging between 600 to 1400bp) and containing one or multiple constrained elements were amplified by PCR (oligo sequences available upon request), cloned in the pGEM-T vector (Promega) and validated by sequencing. Luciferase reporter constructs were generated by subcloning each PCR fragment, in both the sense and antisense orientation, into a modified pXP2 vector containing the 109bp *Thymidine kinase* minimal promoter [61]. Neuro-2a neuroblastoma cells were propagated in EMEM supplemented with 10% FBS whereas P19 embryocarcinoma cells were propagated in alpha-MEM supplemented with 2.5% FBS and 7.5% CBS. Cos7 and NIH 3T3 cells were propagated in DMEM supplemented with 10% FBS. Transfections in 24-well plates and luciferase assays were performed in triplicate at least three times as previously described [62].

### Western blots

Western blots using whole cell extracts of transfected Cos7 cells were performed as previously described [63]. Cells were transfected with a CMVp-driven expression vector for the same Fam162b-IRES-eGFP bicistronic cassette used to produce transgenic embryos and protein expression was assessed using the following primary antibodies: rabbit anti-Fam162b (1:1000; Abcam ab122309), rabbit anti-GFP (1:5000; Abcam ab290) and rabbit anti-Gapdh (1:2500, Santa Cruz Biotech. sc-25778).

### Semi-quantitative RT-PCR

Total RNA was extracted using the RNAeasy Plus purification mini kit (QIAGEN) on FACS-sorted eNCC. The OneStep RT-PCR kit (QIAGEN) was then used on 100 ng of RNA with primers specific to the desired target (sequences available upon request). PCR consisted of 25–30–35 or 30–35–40 cycles of: 20 seconds at 95°C, 30 seconds at 62°C and 30 seconds at 72°C. Amplicons were resolved on a 2% agarose gel and quantified using the densitometry tools of ImageJ. The expression level of the housekeeping gene *Gapdh* was used for normalization.

### Chromosome conformation capture (3C)

3C was performed as previously described [27] with minor modifications. Starting material was either 1x10^8^ cells (for Neuro-2a and P19 cell lines), as recommended, or ~1x10^6^ cells (for e12.5 intestinal material), in which case reaction volumes were divided by a factor of 20. Only one cycle of phenol, then phenol/chloroform extraction was performed. Glycogen (0.05 mg/ml) was added as a co-precipitant prior to ethanol precipitation. For each PCR reaction, 20 ng (BAC library), 50 ng (embryonic intestines libraries) or 100 ng (cell lines libraries) of library DNA was used. Sequencing of the amplicon confirmed the identity of the chimeric fragment amplified.

### Statistics

Data are presented as mean ± standard deviation, with the number of experiments (n) included in the figure and/or legend. Quantification data were subjected to Student's t-test for statistical significance, except for directionality circular data which were compared through an ANOVA. Differences were considered statistically significant when the *p* value was less than 0.05.

## Supporting Information

S1 Fig Fluorescent labelling of NCC in TashT animals. **(a)** The pSRYp[1.6kb]-YFP transgene (co-injected with the *Tyr* minigene) provides fluorescent labelling of migratory NCC in the whole developing embryo, as shown here at stages e10.5, e11.5 and e12.5. Arrowhead shows the track of vagal NCC entering the digestive tract at e10.5. A vibratome transverse section (150 mm thick) of an e11.5 embryo shows extensive YFP fluorescence along NCC migration routes. DRG, dorsal root ganglia; SG, sympathetic ganglia; eNCC, enteric neural crest cells. Note the correspondence between fluorescence intensity and TashT transgene copy number: homozygous e12.5 embryos display twice as much fluorescence than their heterozygous siblings. **(b, c)** Endogenous YFP allows for complete visualization of the forming enteric neural network in TashT embryos. **(b)** Z-stack projection of 133 confocal slices through the midgut wall of an e12.5 TashT embryo showing co-localization of YFP with neuronal (βIII-Tubulin, TuJ1-like) or enteric progenitor/glial (Sox10) markers. As most evidenced by the lateral view of the z-axis in the lower right panel, YFP fluorescence is exclusively restricted to eNCC in this region. **(c)** Z-stack projection of eNCC at the migration front in the proximal hindgut of an e12.5 TashT embryo showing co-expression of YFP and the canonical eNCC marker Ret.
(TIF)

S2 Fig NCC and their derivatives are fluorescently-labeled by both the pSRYp[1.6kb]-YFP and Gata4p[5kb]-GFP transgenes. Confocal projections of various tissues from a TashT::G4-GFP double heterozygote e14.5 embryo. **(a)** Dorsal root ganglion. Most NCC are marked by both transgenes. Empty arrowhead shows a cell expressing only GFP. **(b)** Forming ENS in the midgut. Most cells of the ENS are marked by both transgenes. Arrowhead shows a cell expressing only YFP while empty arrowhead shows a cell expressing only GFP. **(c)** Enteric NCC migration front in the hindgut. Arrowhead shows a mesenchymal cell (not part of the ENS) expressing only YFP. Mesenchymal fluorescence occurs mostly in the cecum and proximal hindgut regions of the TashT intestine (see Fig 2a). Bar: 50 microns.
(TIF)

S3 Fig Morphology of TashT^Tg/Tg^ eNCC. Confocal stack projections of eNCC at the migration front of e11.0 embryos. Normal extended filopodia (arrows) are seen in TashT^Tg/Tg^ eNCC. Note that the group of cells in the TashT^Tg/Tg^ embryo are not entirely isolated, but connected to a migration arm by a thin cytoplasmic bridge (arrowhead). Bar: 50 microns.
(TIF)

S4 Fig Migration paths of leading eNCC in TashT embryos. Five to eight eNCC from the intestines of e11.0 embryos (TashT^Tg/+^ and TashT^Tg/Tg^, 3 intestines for each) were tracked for a minimum of 3 hours before their entry into the cecum in order to quantify migration speed and directionality.
(TIF)

S5 Fig Quantification of proliferation and apoptosis in TashT embryonic intestines. Cell proliferation (**a**) and cell death (**b**) were evaluated in the proximal and distal small intestine (SI) of TashT e12.5 embryos using nucleic markers for proliferation (Ki67) and DNA fragmentation (TUNEL). Nuclei counts were limited to the YFP-labelled NCC in TashT intestines and normalized according to the total number of YFP-positive cells or the surface area they cover (micron^2^). Although none of the variations were statistically significant according to a t-test, cell death tended to be slightly reduced in the proximal SI of TashT^Tg/Tg^ embryos.
(TIF)

S6 Fig Neuronal and glial differentiation of TashT eNCC. **(a, c)** Single confocal slices of e12.5 or e15.5 embryonic small intestines (SI) stained for neuronal (βIII-Tubulin, Tuj1-like; green in **a**) and glial (S100β; green in **c**), as well as enteric progenitor/glial (Sox10; red) markers. Arrowheads point to examples of cell bodies expressing βIII-Tubulin and S100β. Axons marked with βIII-Tubulin are not yet well formed in the distal small intestine of e12.5 embryos. Empty arrowheads (in **c**) point to cell bodies only expressing Sox10. Note that βIII-Tubulin and Sox10 expression is mutually exclusive, while all S100β^+^ glial cells also express Sox10. **(b)** Quantification shows no significant difference in neuronal differentiation between TashT^Tg/+^ and TashT^Tg/Tg^ e12.5 embryonic intestines. Neuronal cells are represented as a percentage of total eNCC (βIII-Tubulin^+^ plus Sox10^+^ cells). **(d)** A marked change in glial differentiation was observed at e15.5 (significant according to a t-test, *: p < 0.05). TashT^Tg/Tg^ embryos possess less glial cells to the profit of undifferentiated progenitors, in both the proximal and distal parts of the small intestine. Glial (S100β^+^, Sox10^+^) and undifferentiated (S100β^-^, Sox10^+^) cells are represented as a percentage of Sox10 expressing cells.
(TIF)

S7 Fig Integration of the TashT transgene on chromosome 10B2/B3. **(a)** FISH analysis using a *Tyrosinase* cDNA probe on Giemsa-stained condensed chromosomes from TashT^Tg/Tg^ cells shows integration of the TashT transgene on chromosome 10 around bands B2–B3 as illustrated on the left. **(b)** Example of the semi-quantitative PCR approach used to determine transgene copy number and genotype TashT animals. Using the oligos depicted on Fig 3a, a non-repeated transgene-specific (Tg) sequence is amplified and normalized to a chromosomic (Chr) amplicon outside of the duplicated region. Band density quantification to determine transgene allele copy number is illustrated below the gel picture.
(TIF)

S8 Fig Expression of genes neighboring the TashT insertion. Semi quantitative RT-PCR of the four genes (two centromeric and two telomeric) flanking the gene desert within which the TashT transgene insertion is found. No differential expression was detected between sorted eNCC from e12.5 control (G4-GFP) and TashT^Tg/Tg^ embryonic guts. *Gapdh* amplification was used as a normalizing control.
(TIF)

S9 Fig Evaluation of transcriptional activity for the 7 cloned regions of the TashT locus in murine embryonic fibroblast (NIH/3T3) and monkey kidney fibroblast (Cos7) cell lines. Luciferase assays were performed with reporter constructs driven by the cloned regions (CE1 to CE7) upstream of a minimal TK promoter. Luciferase activity is reported in fold induction relative to the empty vector (V) which is only driven by the TK minimal promoter. +/- symbols indicate sense and antisense orientation of the cloned fragments in relation to the reporter gene.
(TIF)

S10 Fig Validation and analysis of transcriptome sequencing results. **(a,b)** Semi-quantitative RT-PCR validation for chosen transcripts whose expression is modulated according to RNA sequencing data. RT-PCR was performed on total RNA from FACS-recovered eNCC of e12.5 control (G4-GFP) and TashT^Tg/Tg^ embryonic guts. Expression levels of candidate genes were quantified by densitometry and normalized to *Gapdh* expression (n = 4 independent RNA batches). **(a)** Genes encoding members of the main signaling pathways previously involved in HSCR. **(b)** Downregulated genes located on chromosome X with prospective role in cell migration. There were no modulated genes on chromosome Y. **(c)** Selected categories from Gene Ontology analysis of genes modulated at least 2-fold in TashT^Tg/Tg^ eNCC (1243 gene dataset; Dataset 1) reveals enrichments for cell adhesion, migration and signaling categories amongst others. Number at the right of each bar indicates the number of genes per category.
(TIF)

S11 Fig *Fam162b* is strongly upregulated in TashT^Tg/Tg^ eNCC and encodes a protein of unknown function. **(a)** Semi quantitative RT-PCR of *Fam162b* on total RNA from FACS-recovered eNCC of e12.5 control (G4-GFP) and TashT^Tg/Tg^ embryonic guts. Expression levels were quantified by densitometry and normalized to *Gapdh* expression (n = 4 independent RNA batches). **(b)** Western blot validation of protein expression from a Fam162b-IRES-eGFP bicistronic cassette in transfected Cos7 cells. This bicistronic cassette was subsequently used to produce transgenic embryos under the control of the *Sox10* U3 enhancer (Fig 4e).
(TIF)

S1 Movie Example of eNCC migration in a e11.0 control embryo. (AVI)

S2 Movie Example of eNCC migration in a e11.0 TashT^Tg/Tg^ embryo. (AVI)

S1 Dataset All 1243 significantly deregulated genes in e12.5 TashT^Tg/Tg^ eNCC. (XLSX)

S2 Dataset Full rRNA-depleted transcriptome of e12.5 G4-GFP and TashT^Tg/Tg^ eNCC. (XLS)
