# Supplementary figures and images for: Male-Biased Aganglionic Megacolon in the TashT Mouse Line Due to Perturbation of Silencer Elements in a Large Gene Desert of Chromosome 10
Source: PLoS Genet. 2015 Mar 18;11(3):e1005093. doi: 10.1371/journal.pgen.1005093 (PMC4364714; doi:10.1371/journal.pgen.1005093)

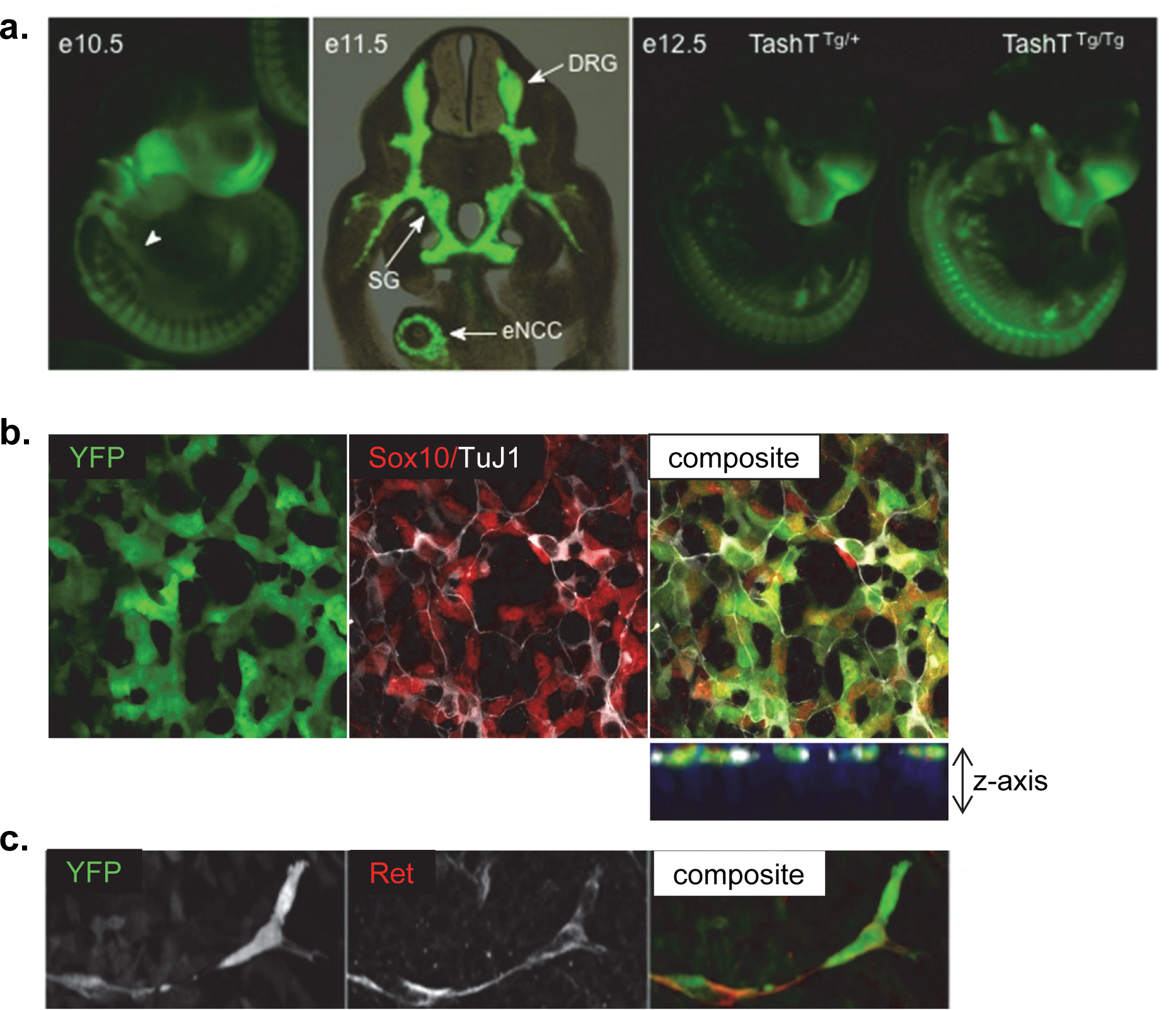

Supplement: S1 Fig — (a) The pSRYp[1.6kb]-YFP transgene (co-injected with the Tyr minigene) provides fluorescent labelling of migratory NCC in the whole developing embryo, as shown here at stages e10.5, e11.5 and e12.5. Arrowhead shows the track of vagal NCC entering the digestive tract at e10.5. A vibratome transverse section (150 mm thick) of an e11.5 embryo shows extensive YFP fluorescence along NCC migration routes. DRG, dorsal root ganglia; SG, sympathetic ganglia; eNCC, enteric neural crest cells. Note the correspondence between fluorescence intensity and TashT transgene copy number: homozygous e12.5 embryos display twice as much fluorescence than their heterozygous siblings. (b, c) Endogenous YFP allows for complete visualization of the forming enteric neural network in TashT embryos. (b) Z-stack projection of 133 confocal slices through the midgut wall of an e12.5 TashT embryo showing co-localization of YFP with neuronal (βIII-Tubulin, TuJ1-like) or enteric progenitor/glial (Sox10) markers. As most evidenced by the lateral view of the z-axis in the lower right panel, YFP fluorescence is exclusively restricted to eNCC in this region. (c) Z-stack projection of eNCC at the migration front in the proximal hindgut of an e12.5 TashT embryo showing co-expression of YFP and the canonical eNCC marker Ret. (TIF) [file pgen.1005093.s001.tif]

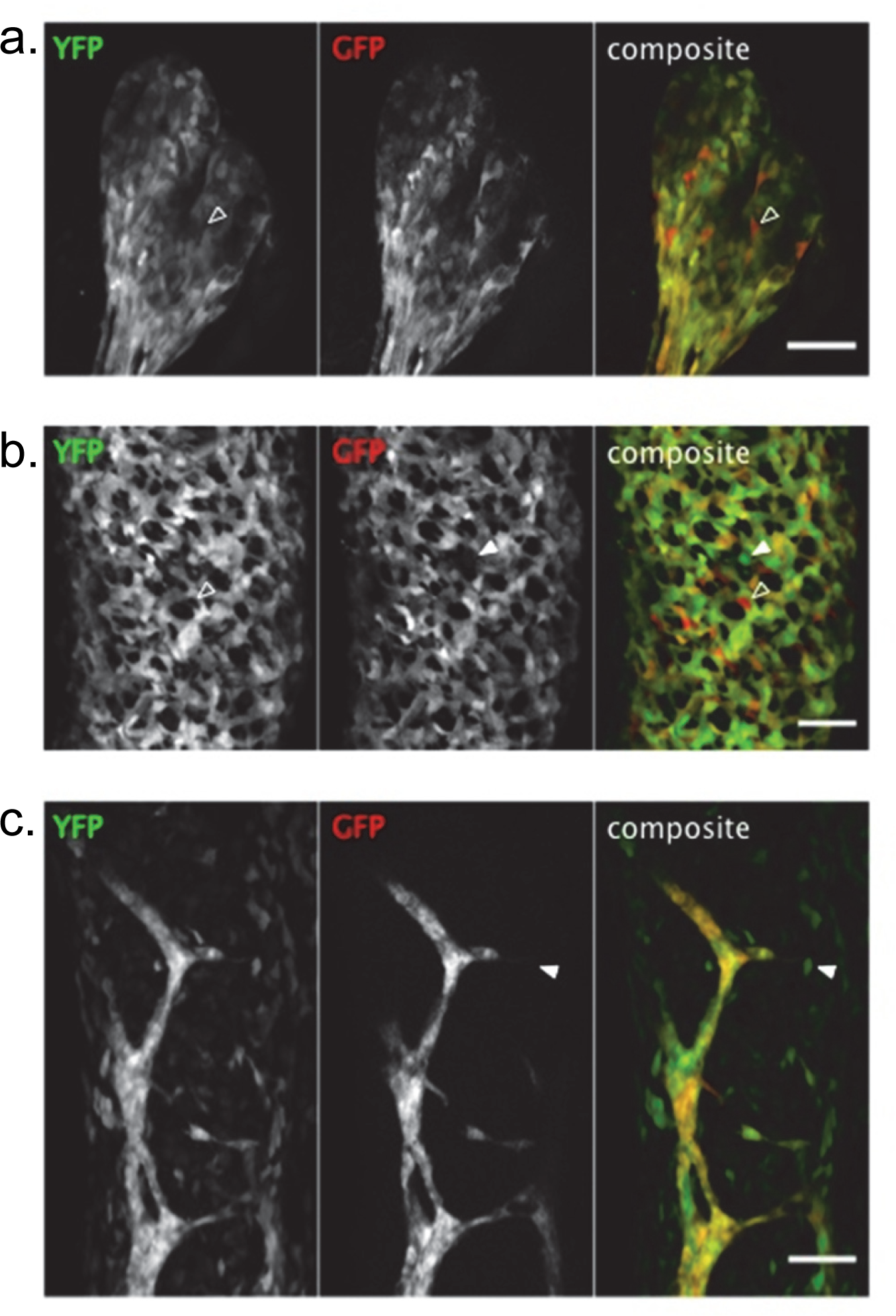

Supplement: S2 Fig — Confocal projections of various tissues from a TashT::G4-GFP double heterozygote e14.5 embryo. (a) Dorsal root ganglion. Most NCC are marked by both transgenes. Empty arrowhead shows a cell expressing only GFP. (b) Forming ENS in the midgut. Most cells of the ENS are marked by both transgenes. Arrowhead shows a cell expressing only YFP while empty arrowhead shows a cell expressing only GFP. (c) Enteric NCC migration front in the hindgut. Arrowhead shows a mesenchymal cell (not part of the ENS) expressing only YFP. Mesenchymal fluorescence occurs mostly in the cecum and proximal hindgut regions of the TashT intestine (see Fig 2a). Bar: 50 microns. (TIF) [file pgen.1005093.s002.tif]

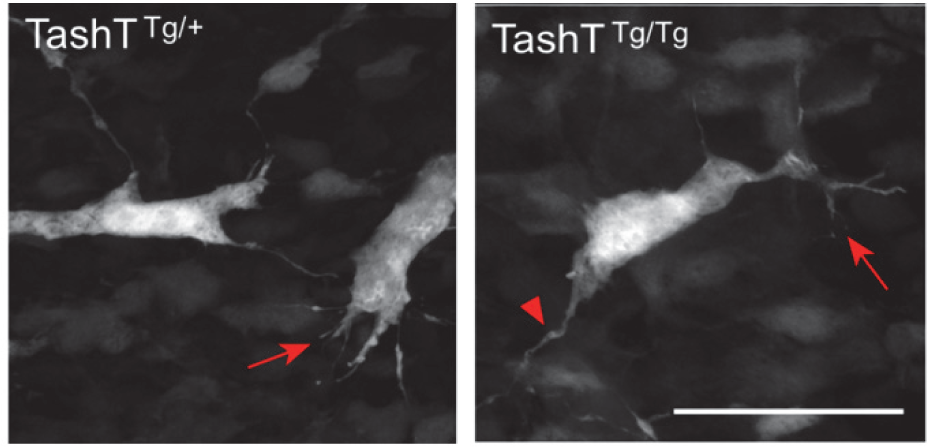

Supplement: S3 Fig — Confocal stack projections of eNCC at the migration front of e11.0 embryos. Normal extended filopodia (arrows) are seen in TashTTg/Tg eNCC. Note that the group of cells in the TashTTg/Tg embryo are not entirely isolated, but connected to a migration arm by a thin cytoplasmic bridge (arrowhead). Bar: 50 microns. (TIF) [file pgen.1005093.s003.tif]

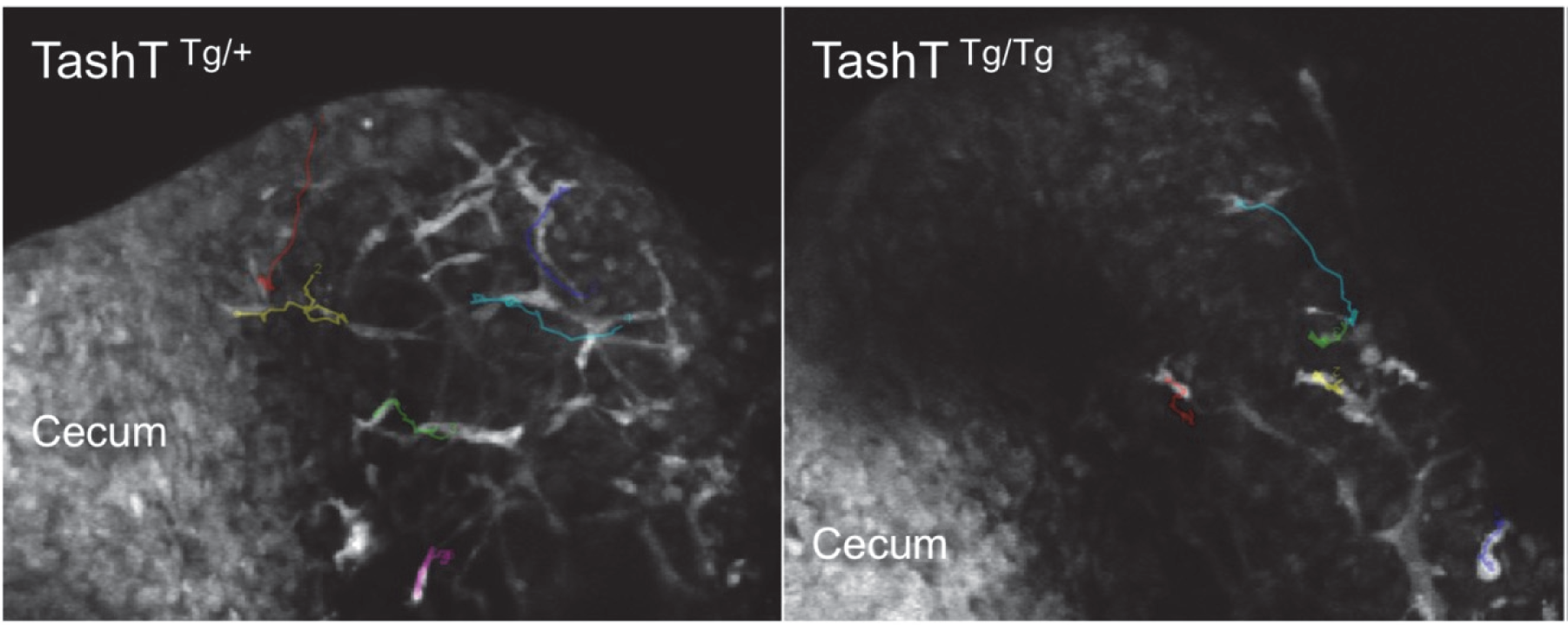

Supplement: S4 Fig — Five to eight eNCC from the intestines of e11.0 embryos (TashTTg/+ and TashTTg/Tg, 3 intestines for each) were tracked for a minimum of 3 hours before their entry into the cecum in order to quantify migration speed and directionality. (TIF) [file pgen.1005093.s004.tif]

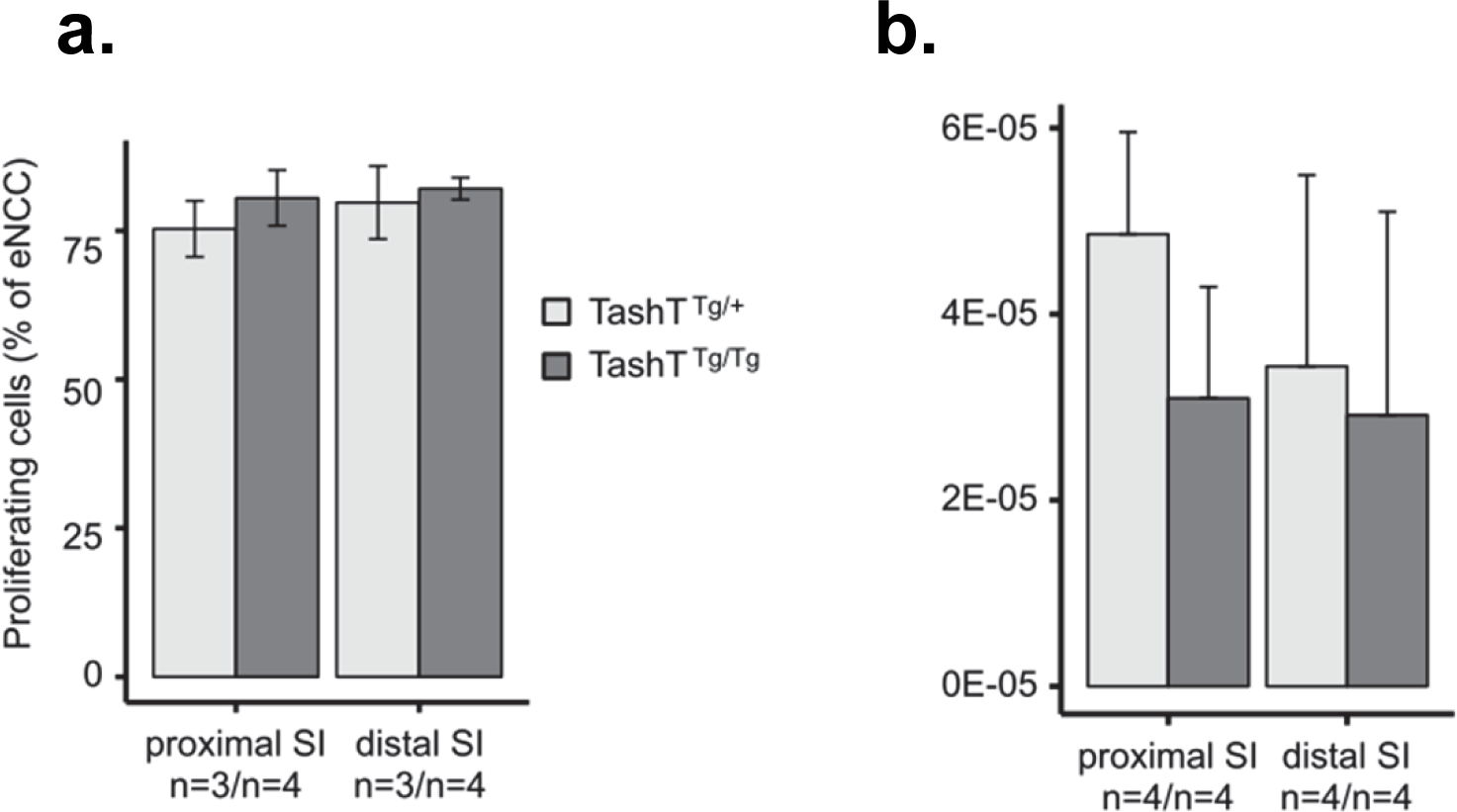

Supplement: S5 Fig — Cell proliferation (a) and cell death (b) were evaluated in the proximal and distal small intestine (SI) of TashT e12.5 embryos using nucleic markers for proliferation (Ki67) and DNA fragmentation (TUNEL). Nuclei counts were limited to the YFP-labelled NCC in TashT intestines and normalized according to the total number of YFP-positive cells or the surface area they cover (micron2). Although none of the variations were statistically significant according to a t-test, cell death tended to be slightly reduced in the proximal SI of TashTTg/Tg embryos. (TIF) [file pgen.1005093.s005.tif]

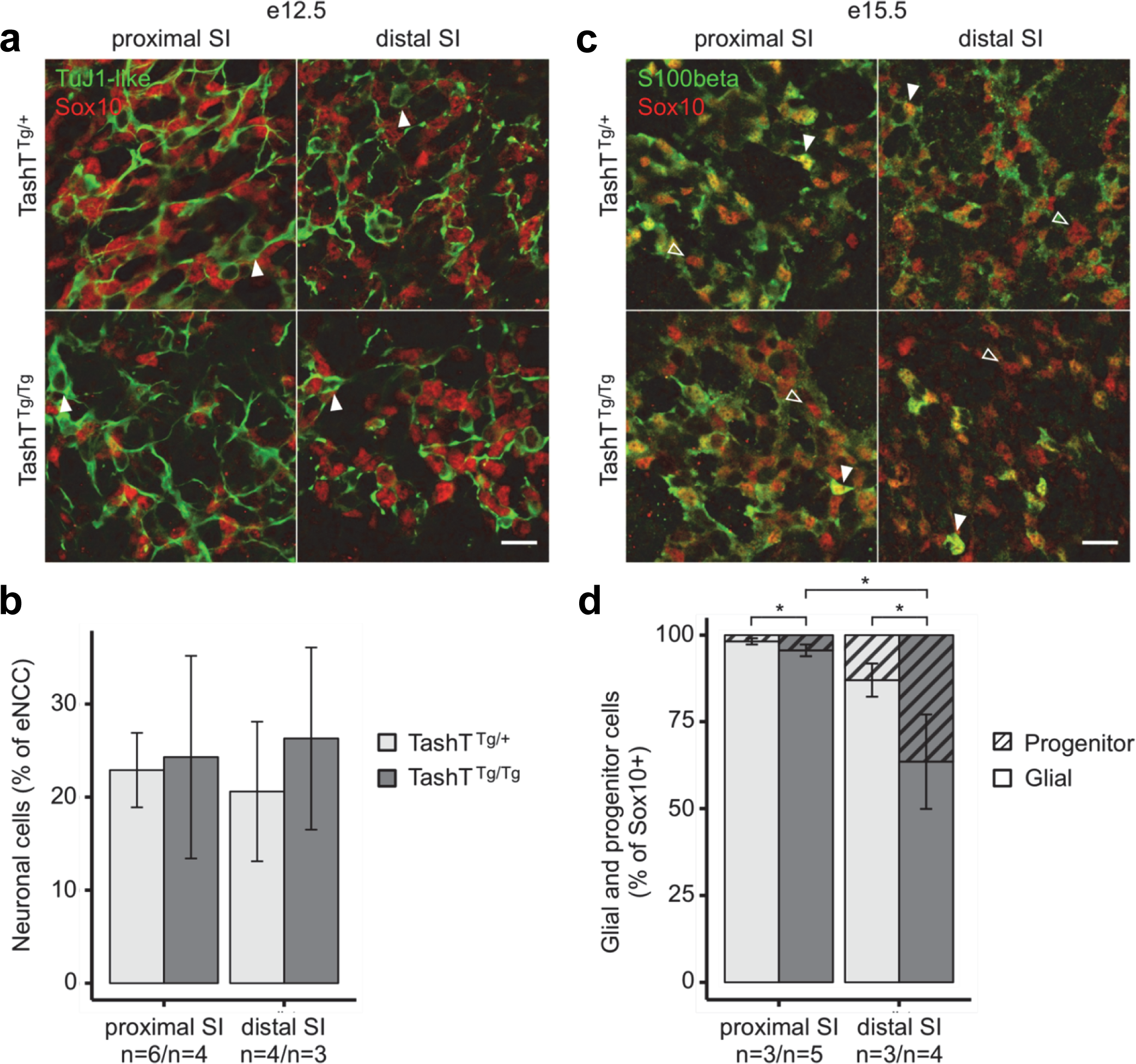

Supplement: S6 Fig — (a, c) Single confocal slices of e12.5 or e15.5 embryonic small intestines (SI) stained for neuronal (βIII-Tubulin, Tuj1-like; green in a) and glial (S100β; green in c), as well as enteric progenitor/glial (Sox10; red) markers. Arrowheads point to examples of cell bodies expressing βIII-Tubulin and S100β. Axons marked with βIII-Tubulin are not yet well formed in the distal small intestine of e12.5 embryos. Empty arrowheads (in c) point to cell bodies only expressing Sox10. Note that βIII-Tubulin and Sox10 expression is mutually exclusive, while all S100β+ glial cells also express Sox10. (b) Quantification shows no significant difference in neuronal differentiation between TashTTg/+ and TashTTg/Tg e12.5 embryonic intestines. Neuronal cells are represented as a percentage of total eNCC (βIII-Tubulin+ plus Sox10+ cells). (d) A marked change in glial differentiation was observed at e15.5 (significant according to a t-test, *: p < 0.05). TashTTg/Tg embryos possess less glial cells to the profit of undifferentiated progenitors, in both the proximal and distal parts of the small intestine. Glial (S100β+, Sox10+) and undifferentiated (S100β-, Sox10+) cells are represented as a percentage of Sox10 expressing cells. (TIF) [file pgen.1005093.s006.tif]

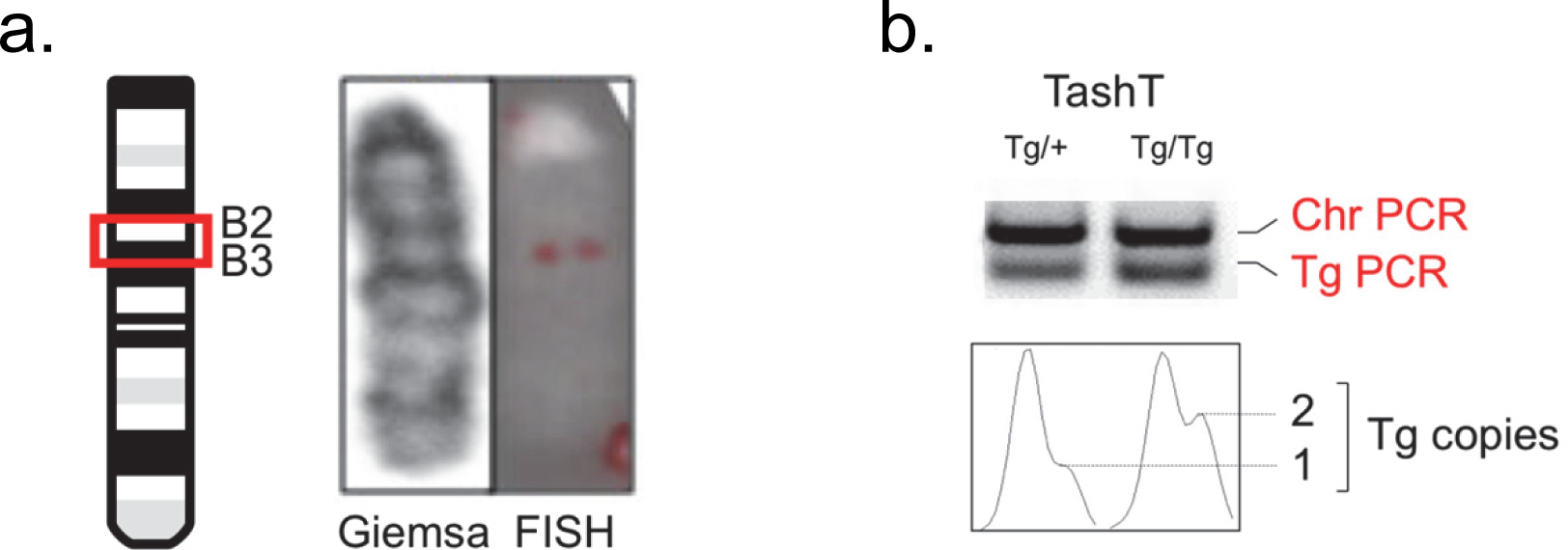

Supplement: S7 Fig — (a) FISH analysis using a Tyrosinase cDNA probe on Giemsa-stained condensed chromosomes from TashTTg/Tg cells shows integration of the TashT transgene on chromosome 10 around bands B2–B3 as illustrated on the left. (b) Example of the semi-quantitative PCR approach used to determine transgene copy number and genotype TashT animals. Using the oligos depicted on Fig 3a, a non-repeated transgene-specific (Tg) sequence is amplified and normalized to a chromosomic (Chr) amplicon outside of the duplicated region. Band density quantification to determine transgene allele copy number is illustrated below the gel picture. (TIF) [file pgen.1005093.s007.tif]

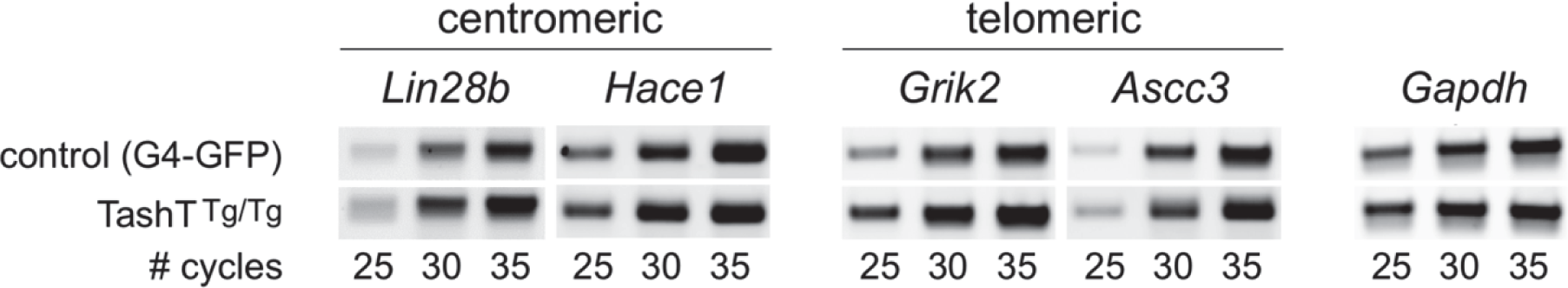

Supplement: S8 Fig — Semi quantitative RT-PCR of the four genes (two centromeric and two telomeric) flanking the gene desert within which the TashT transgene insertion is found. No differential expression was detected between sorted eNCC from e12.5 control (G4-GFP) and TashTTg/Tg embryonic guts. Gapdh amplification was used as a normalizing control. (TIF) [file pgen.1005093.s008.tif]

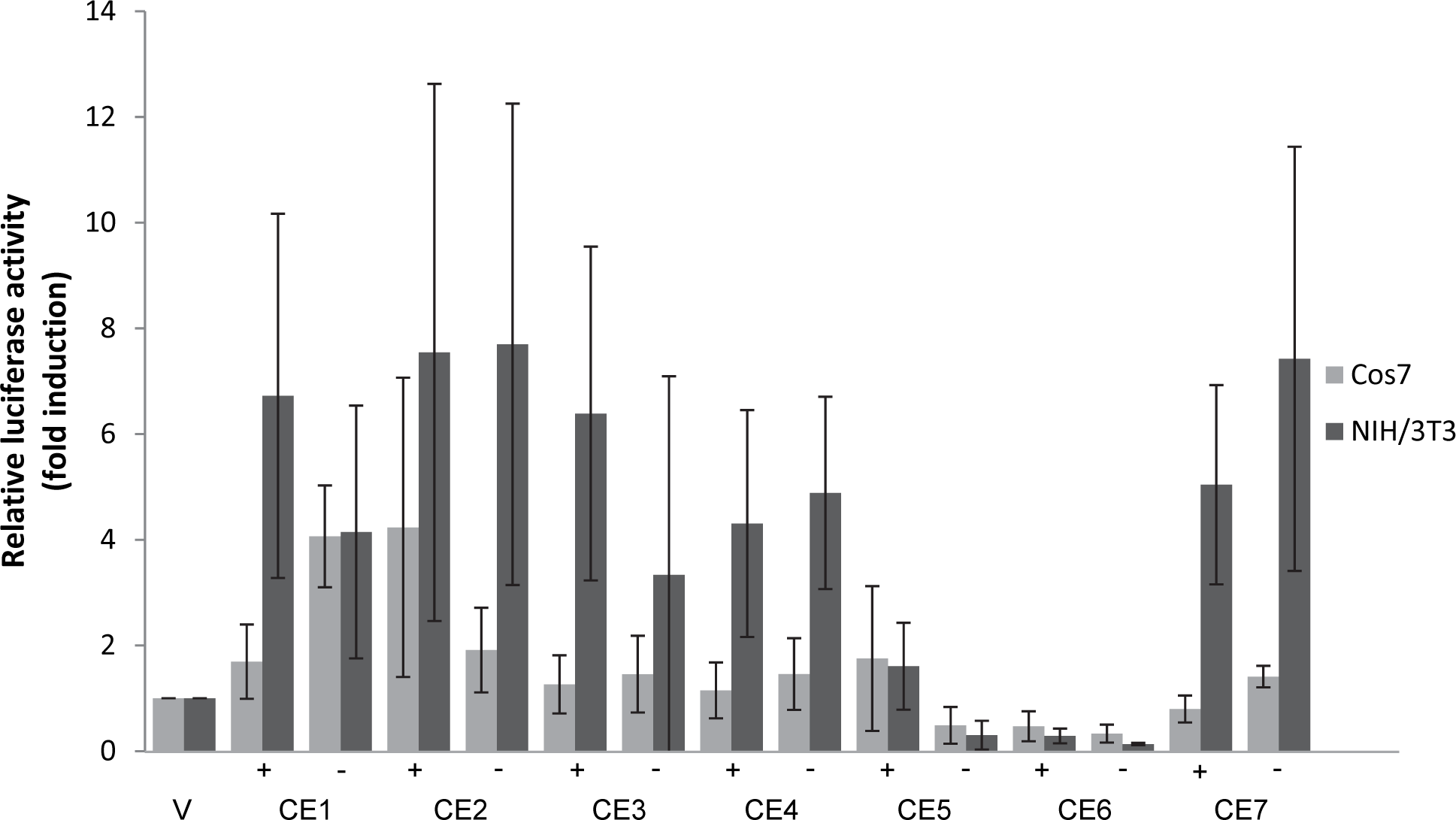

Supplement: S9 Fig — Luciferase assays were performed with reporter constructs driven by the cloned regions (CE1 to CE7) upstream of a minimal TK promoter. Luciferase activity is reported in fold induction relative to the empty vector (V) which is only driven by the TK minimal promoter. +/- symbols indicate sense and antisense orientation of the cloned fragments in relation to the reporter gene. (TIF) [file pgen.1005093.s009.tif]

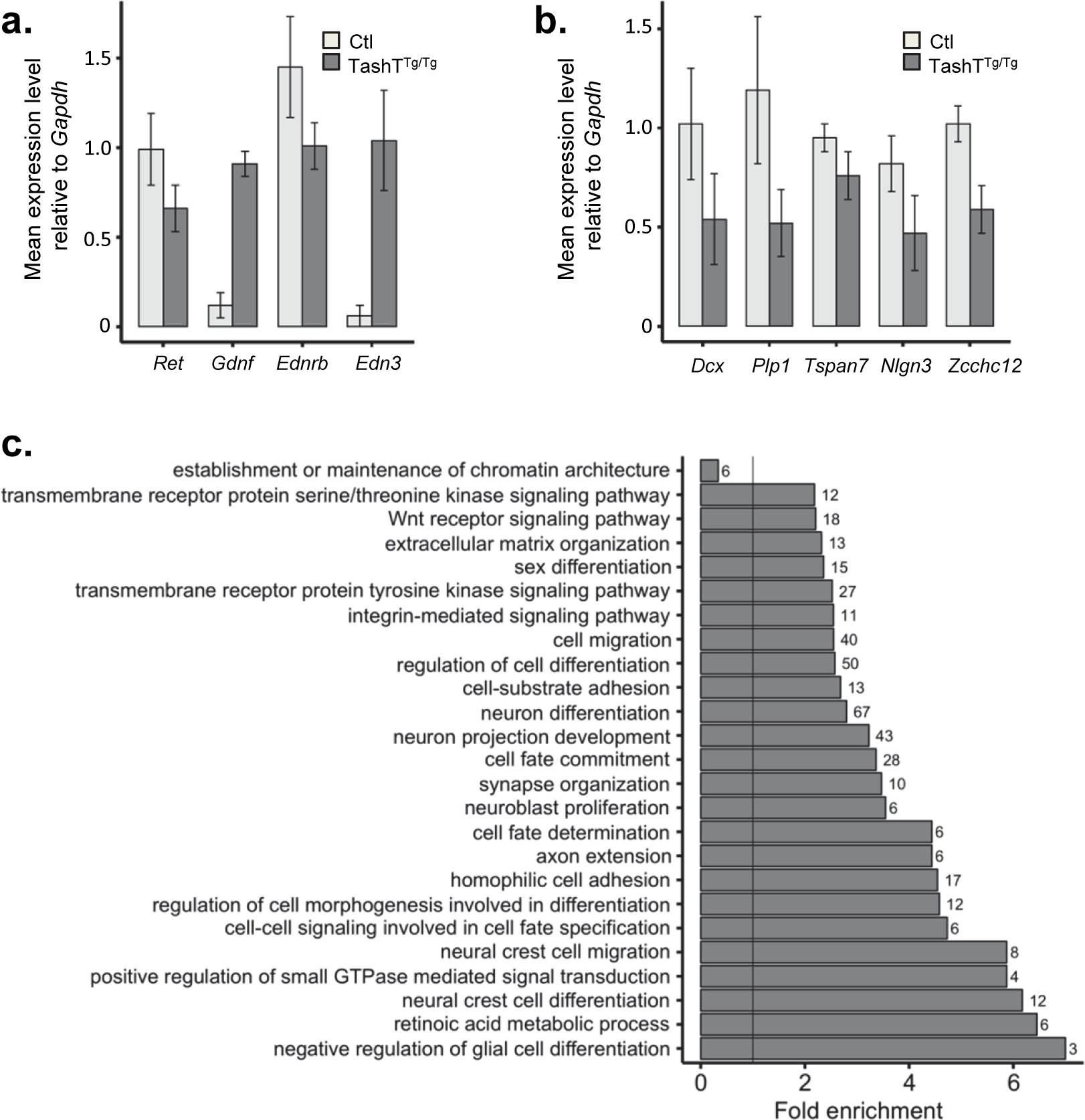

Supplement: S10 Fig — (a,b) Semi-quantitative RT-PCR validation for chosen transcripts whose expression is modulated according to RNA sequencing data. RT-PCR was performed on total RNA from FACS-recovered eNCC of e12.5 control (G4-GFP) and TashTTg/Tg embryonic guts. Expression levels of candidate genes were quantified by densitometry and normalized to Gapdh expression (n = 4 independent RNA batches). (a) Genes encoding members of the main signaling pathways previously involved in HSCR. (b) Downregulated genes located on chromosome X with prospective role in cell migration. There were no modulated genes on chromosome Y. (c) Selected categories from Gene Ontology analysis of genes modulated at least 2-fold in TashTTg/Tg eNCC (1243 gene dataset; Dataset 1) reveals enrichments for cell adhesion, migration and signaling categories amongst others. Number at the right of each bar indicates the number of genes per category. (TIF) [file pgen.1005093.s010.tif]

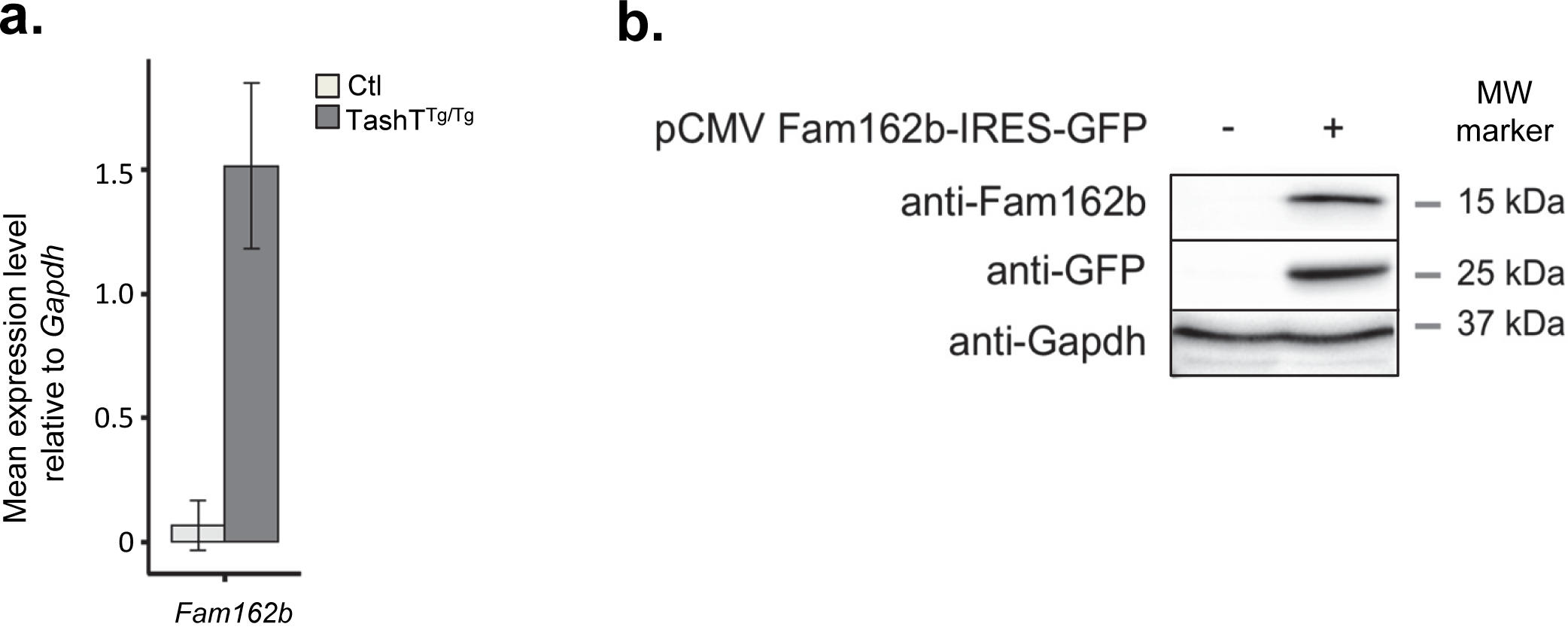

Supplement: S11 Fig — (a) Semi quantitative RT-PCR of Fam162b on total RNA from FACS-recovered eNCC of e12.5 control (G4-GFP) and TashTTg/Tg embryonic guts. Expression levels were quantified by densitometry and normalized to Gapdh expression (n = 4 independent RNA batches). (b) Western blot validation of protein expression from a Fam162b-IRES-eGFP bicistronic cassette in transfected Cos7 cells. This bicistronic cassette was subsequently used to produce transgenic embryos under the control of the Sox10 U3 enhancer (Fig 4e). (TIF) [file pgen.1005093.s011.tif]
